# Connexin 43 confers chemoresistance through activating PI3K

**DOI:** 10.1038/s41389-022-00378-7

**Published:** 2022-01-12

**Authors:** Kevin J. Pridham, Farah Shah, Kasen R. Hutchings, Kevin L. Sheng, Sujuan Guo, Min Liu, Pratik Kanabur, Samy Lamouille, Gabrielle Lewis, Marc Morales, Jane Jourdan, Christina L. Grek, Gautam G. Ghatnekar, Robin Varghese, Deborah F. Kelly, Robert G. Gourdie, Zhi Sheng

**Affiliations:** 1 Fralin Biomedical Research Institute at VTC, Roanoke, VA 24016 USA; 2 Department of Internal Medicine, Virginia Tech Carilion School of Medicine, Roanoke, VA 24016 USA; 3 Department of Biomedical Affairs and Research, Edward Via College of Osteopathic Medicine, Blacksburg, VA 24060 USA; 4 Department of Basic Science Education, Virginia Tech Carilion School of Medicine, Roanoke, VA 24016 USA; 5 Department of Biological Sciences, Virginia Tech, Blacksburg, VA 24061 USA; 6 FirstString Research, Inc, Mount Pleasant, SC 29464 USA; 7 Department of Biomedical Engineering, Pennsylvania State University, University Park, PA 16802 USA; 8 Huck Institutes of the Life Sciences, Pennsylvania State University, University Park, PA 16802 USA; 9 Center for Structural Oncology, Pennsylvania State University, University Park, PA 16802 USA; 10 Department of Emergency Medicine, Virginia Tech Carilion School of Medicine, Roanoke, VA 24016 USA; 11 Faculty of Health Science, Virginia Tech, Blacksburg, VA 24061 USA

**Keywords:** CNS cancer, Target validation, Phosphoinositol signalling, Cancer therapeutic resistance

## Abstract

Circumventing chemoresistance is crucial for effectively treating cancer including glioblastoma, a lethal brain cancer. The gap junction protein connexin 43 (Cx43) renders glioblastoma resistant to chemotherapy; however, targeting Cx43 is difficult because mechanisms underlying Cx43-mediated chemoresistance remain elusive. Here we report that Cx43, but not other connexins, is highly expressed in a subpopulation of glioblastoma and Cx43 mRNA levels strongly correlate with poor prognosis and chemoresistance in this population, making Cx43 the prime therapeutic target among all connexins. Depleting Cx43 or treating cells with αCT1–a Cx43 peptide inhibitor that sensitizes glioblastoma to the chemotherapy temozolomide–inactivates phosphatidylinositol-3 kinase (PI3K), whereas overexpression of Cx43 activates this signaling. Moreover, αCT1-induced chemo-sensitization is counteracted by a PI3K active mutant. Further research reveals that αCT1 inactivates PI3K without blocking the release of PI3K-activating molecules from membrane channels and that Cx43 selectively binds to the PI3K catalytic subunit β (PIK3CB, also called PI3Kβ or p110β), suggesting that Cx43 activates PIK3CB/p110β independent of its channel functions. To explore the therapeutic potential of simultaneously targeting Cx43 and PIK3CB/p110β, αCT1 is combined with TGX-221 or GSK2636771, two PIK3CB/p110β-selective inhibitors. These two different treatments synergistically inactivate PI3K and sensitize glioblastoma cells to temozolomide in vitro and in vivo. Our study has revealed novel mechanistic insights into Cx43/PI3K-mediated temozolomide resistance in glioblastoma and demonstrated that targeting Cx43 and PIK3CB/p110β together is an effective therapeutic approach for overcoming chemoresistance.

## Introduction

Overcoming resistance to chemotherapy such as temozolomide (TMZ) has proven perplexing and remains a key unmet clinical need. As an alkylating agent, TMZ reacts with DNA at multiple sites, yielding O^6^-methylguanine lesions that subsequently induce DNA breaks and eventually cell death [1]. Given that TMZ is able to pass the blood–brain barrier [2], this drug has been used as the frontline chemotherapy for glioblastoma (GBM) an aggressive and lethal cancer that accounts for approximately half of all malignant brain tumors and has a grim prognosis with an average survival time of 14.6 months [3, 4]. Adding to this dismal outcome, nearly 90% of patients with GBM succumb to tumor recurrence and the average survival for recurrent GBM is about 5.5–7.5 months due to limited therapeutic options and resistance to TMZ [5]. Hence, overcoming TMZ resistance is key to effectively treating GBM and curbing GBM progression. Poor responses of nearly 50% of GBM patients to TMZ are due to the expression of O-6-methylguanine-DNA methyltransferase (MGMT) [6, 7]. MGMT repairs TMZ-induced DNA damage, conferring MGMT-dependent TMZ resistance; as such, inhibiting MGMT has shown encouraging clinical benefits [8]. Patients with no MGMT expression also develop MGMT-independent resistance to TMZ [9, 10]. Factors involved in MGMT-independent TMZ resistance include the DNA mismatch repair pathway and genetic alterations [11, 12]. However, targeting these factors to circumvent TMZ resistance has been a daunting task. Deeper insights into MGMT-independent TMZ resistance are therefore needed.

Recently, several lines of evidence have indicated that the gap junction protein connexin 43 (Cx43; also known as gap junction protein A1, *GJA1*), a channel-forming protein important for intercellular communication [13], controls the response of GBM cells to TMZ. Ectopic expression of Cx43 renders GBM cells resistant to TMZ [14–17], and blocking Cx43 using different approaches such as antibodies or channel inhibitors restores TMZ sensitivity [14–20]. However, it remains unclear whether Cx43-mediated TMZ resistance depends on MGMT. Our recent work [21] reveals that high levels of Cx43 in MGMT-deficient GBM cell lines and primary patient samples correlate with poor responses to TMZ and that αCT1, a clinically-tested therapeutic peptide that comprises the Cx43 carboxyl terminus (CT) and an antennapedia cell-penetrating sequence [22], antagonizes TMZ resistance. Nonetheless, the molecular underpinnings of Cx43-mediated TMZ resistance remain elusive, making it difficult to effectively target Cx43 to treat GBM.

In this report, we determined the role of connexins in GBM prognosis and TMZ resistance, explored how Cx43 activates phosphatidylinositol-3 kinase (PI3K) independent of Cx43 channels and induces TMZ resistance, and examined a candidate triple combinational therapy entailing the Cx43 inhibitor αCT1, PI3K-selective inhibitors, and TMZ in preclinical studies for its effectiveness in overcoming TMZ resistance.

## Materials and methods

### Reagents

Resources and catalog numbers of reagents were included in Supplemental Table S1. Chemical compounds were reconstituted in dimethyl sulfoxide (DMSO) at a concentration of 50–80 mM. Peptide αCT1 and Gap27 were in vitro synthesized and purchased from LifeTein, LLC. Lyophilized peptide was reconstituted in 1x PBS (137 mM NaCl, 2.7 mM KCl, 10 mM Na_2_HPO_4_, and 1.8 mM KH_2_PO_4_) at a concentration of 5 or 10 mM. All chemicals were aliquoted and stored at -80 °C.

### Cell culture

GBM cell lines, primary GBM cells, GBM stem cells (GSCs), and human astrocytes were cultured as previously described [21, 23–30]. Cell lines have been authenticated by the ATCC authentication service utilizing Short Tandem Repeat profiling and tested for mycoplasma contamination. Primary cells VTC-001, VTC-003, VTC-005, and VTC-103 were cultured in DMEM supplemented with 15% fetal bovine serum (Peak Serum, Inc.) and penicillin/streptomycin. Normal human astrocytes were cultured in MCDB-131 medium (Sigma) containing 3% fetal bovine serum (Peak Serum, Inc.), 10x G-5 Supplement (Gibco), and penicillin/streptomycin. Primary GBM cells were kept at low passages (no more than 10).

### Analysis of online databases

Analysis of online databases has been described previously [21, 26–29]. Transcriptomic and proteomic datasets with corresponding clinical information and immunostaining data of human tissues are downloaded from the following websites: (1) The Cancer Gene Atlas (TCGA) datasets: https://www.cbioportal.org and https://gliovis.shinyapps.io/GlioVis/; (2) Gravendeel, Rembrandt, Lee Y, and Murat GBM: https://gliovis.shinyapps.io/GlioVis/; (3) The China Glioma Gene Atlas (CGGA) datasets: https://gliovis.shinyapps.io/GlioVis/; (4) GBM cell lines from the Cancer Dependency Map (DepMap): https://depmap.org/portal/; (5) The Human Protein Atlas: https://www.proteinatlas.org. The Kaplan–Meier survival analysis or the Cox hazard proportional model were used to determine the association of gene expression and patient survival. The Pearson correlation coefficient, calculated using Prism 9 software, was used to determine the expression correlation between different genes/proteins, The histologic images from The Human Protein Atlas were quantified using Image J and further analyzed using Prism 9.

### MTS cell viability assay

Cell viability was determined by the MTS cell viability assay as described previously [21, 23, 26–35]. In brief, 250–1000 cells were plated in the wells of a 96-well plate. Cells were then treated with DMSO, chemical inhibitors, or αCT1 peptides at the indicated doses for 6 days. For αCT1 treatment, we intended to plate cells at a low density to minimize the formation of gap junctions, and thus, more Cx43-hemichannels will be present. Because the half-life of αCT1 is about 48 h, cells were replenished with fresh peptide every other day. MTS reagent was added and cell viability was determined by measuring the absorbance at 490 nm (MTS) using a FilterMax F3 microplate reader (Molecular Devices, LLC). Percent cell viability was obtained by dividing the absorbance of treatment groups to those of untreated and respective vehicle control groups.

### Caspase 3/7 activity assay

Apoptosis was measured using the Caspase-Glo^®^ 3/7 Assay (Promega) based on the manufacturer’s instructions and our previous work [21, 27–29]. In brief, cells were plated at 250-1000 cells/well in 96-well plates and treated with drugs as described for 6 days. Caspase-Glo^®^ reagent was added and the luminescence was measured using a FilterMax F3 microplate reader.

### Immunoblotting

Immunoblotting was performed as described previously [21, 23, 26–29, 31, 32, 35–38]. To prepare cell lysates from tumor tissues, lysis buffer (20 mM HEPES pH 6.8, 140 mM NaCl, 2.5 mM MgCl_2_, 2.5 mM CaCl_2_, 1% NP40, 0.5% sodium deoxycholate, protease inhibitor, and phosphatase inhibitors) was added to minced tissues followed by homogenization and protein quantification. 25–50 μg total proteins from cell lysates were resolved on SDS-PAGE gels and transferred onto PVDF membranes. Antibodies were added and their dilution rates were summarized in Supplemental Table S1. Images of chemiluminescence from SuperSignal West Pico or Femto substrates (ThermoFisher) were taken using the ChemiDoc MP (BioRad). Whole images of immunoblotting were presented in the Supplemental Data-Whole blot images.

### Co-immunoprecipitation

Co-immunoprecipitation was performed as previously described [37]. Cell pellets were lysed in lysis buffer described above. Total protein lysates were divided equally for each IP with input and IgG controls. Samples were incubated with primary antibodies overnight at 4 °C followed by Protein G Dynabeads. Antibodies and IgG dilutions were listed in Supplemental Table S1. Dilution rates vary due to recommendations from manufacturers and different concentrations of antibodies and IgG.

### Gene knockdown or expression

Knockdown of Cx43 or PI3K genes was described previously [21, 26, 27]. Information regarding short hairpin (sh) RNA of Cx43 or PI3K genes or constructs expressing gene mutants was included in the Supplemental Table S1. pcDNA3.2-GJA1 STOP was kindly provided by Dr. James Smyth at the Fralin Biomedical Research Institute (Roanoke, Virginia). In brief, lentiviral or retroviral vectors containing shRNAs or gene mutants were used to transfect HEK293T cells to generate viruses. Lentiviruses or retroviruses were subsequently used to transduce tumor cells. For transient transfection, 1–2 μg of plasmid DNA was transfected into cells using Effectene (QIAGEN). Transduced or transfected cells were subject to analyses of immunoblotting as described above.

### ATP/glutamate release

ATP release was measured using the Kinase-Glo^®^ Luminescent Kinase Assay (Promega). Glutamate release was measured using the Amplex™ Red Glutamic Acid/Glutamate Oxidase Assay Kit (ThermoFisher). In brief, 1000 cells were plated in the wells of 96-well plates followed by the indicated treatments. Media and cell lysates were then analyzed using the above kits according to the manufacturer’s instructions.

### Bliss independence model

Synergism was assessed by Bliss independence [39, 40], which is based on the null hypothesis that each drug acts independently without super-additive or antagonistic effects. The predicted effect was calculated using Effect(a + b) = Ea+Eb−EaEb for dual therapies and Effect(a + b + c) = Ea+Eb+Ec–EaEb–EaEc–EbEc−EaEbEc for triple therapies. The overall effect was presented as Excess Over Bliss (EOB) scores. EOB > 0% indicates a synergistic effect, whereas EOB = 0% indicates an additive effect and EOB < 0% indicates an antagonistic effect.

### Mouse experiments

Mouse experiments were performed based on the methods described previously [27, 29, 38], with modifications. All animal studies were approved by the Institutional Animal Care and Use Committee of Virginia Tech. In total, 2 × 10^6^ SF-295 cells were mixed with Matrigel^®^ Matrix (Corning) and subcutaneously injected into the flanks of 8-week-old SCID/beige mice (Taconic Biosciences). As we were measuring tumor sizes at different time points, 8 mice were assigned to each treatment group 8 days post-injection. The sample size was determined based on power analysis using G*Power. Mice were treated with drugs as indicated in the figure. No blinding was used. Drugs were administered every other day via intraperitoneal injection (TMZ and TGX-221) or through intratumoral injection (αCT1). Tumors were measured daily using a caliper. Sick mice or mice with 10% weight loss would be excluded. On day 18, mice were euthanized, and tumors were harvested. Tumor volumes (mm^3^) were calculated using the formula: (length × width^2^)/2.

### Statistical analyses

Distribution and variation of data points in each experiment were analyzed and estimated. Three to four independent experiments were performed in cell-based assays, yielding standard deviations shown as error bars. Drug combinations were repeated in different cell lines. One-way ANOVA with Dunnett test for correction of multiple comparisons, Fisher’s exact test, and Student’s *t* test were used to determine statistical significance and similarity of variance. All tests were two-sided. The center values shown in each figure were means.

## Results

### Cx43, but not other connexins, is highly expressed in a subpopulation of GBM and mRNA levels of Cx43 correlate with poor prognosis and chemoresistance

**Fig. 1:**
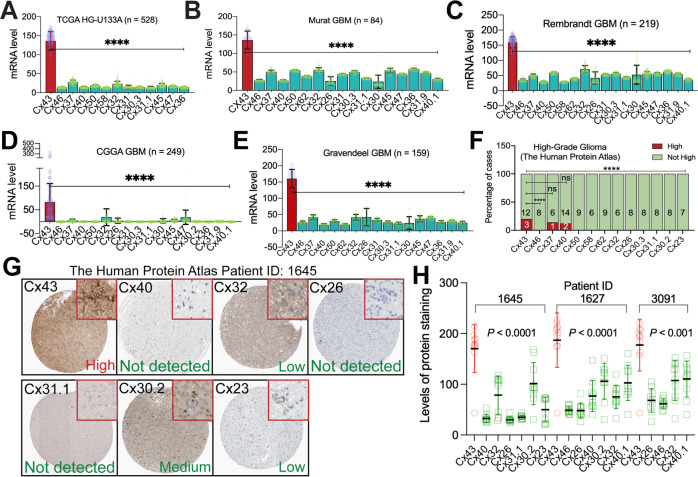
Cx43 is expressed at the highest level among all connexins in a subpopulation of GBM. mRNA levels of connexins in GBMs from TCGA (**A**), Murat (**B**), Rembrandt (**C**), CGGA (**D**), and Gravendeel (**E**). Shown are average reads of microarray or RNAseq. Cx43 is presented as red bars with purple data points. Other connexins are labeled as green bars and yellow data points. **F** Staining scores of connexins in high-grade glioma. Case numbers with high (red) or not high (green) levels of connexins are shown. **G** Histological images of connexins in a high-grade glioma tumor. Inset images (highlighted in red) were cropped from original images in order to highlight immunostaining details. **H** Quantification of histological images from three GBM patients using Imaging J. Intensities of protein staining in 10 different cells were measured and quantified. GBM datasets were retrieved from cBioPortal, GlioVis, or CGGA data portal. Immunostaining results of high-grade glioma were obtained from the Human Protein Atlas. Statistical analyses: One-Way ANOVA with Dunnett test for correction of multiple comparisons and Fisher’s exact test. ns not significant; ****P < 0.0001. Error bars are standard deviations.

Studies on the expression of Cx43 in normal brain and malignant glioma have shown discrepant results [15, 41–48]. Despite that Cx43 mRNA levels are usually high in normal brain and low-grade glioma and vary in high-grade glioma, whether Cx43 and other connexins (Supplemental Table S2) are equally expressed in GBM and important for GBM chemoresistance has not yet been explored. To address this, we queried publicly available online GBM databases and analyzing programs, including TCGA (https://www.cancer.gov/tcga), GlioVis [49], CGGA [50], and DepMap [51]. Cx43 mRNA was consistently expressed at the highest level among all connexins in primary GBM tumors from six different datasets (sample size from 84 to 526) and 54 GBM cell lines (Fig. 1A–E and Supplemental Fig. S1; *P* < 0.0001). Based on immunostaining results from The Human Protein Atlas [52], levels of Cx43 protein in high-grade glioma were higher than other connexins, except Cx37 or Cx40 (Fig. 1F). In the same GBMs expressing high levels of Cx43 (Cx43-high), staining intensities of Cx43 were the highest compared to other connexins (Fig. 1G, H and Supplemental Fig. S2). This expression pattern was not found in low-grade glioma (Supplemental Fig. S3A). Intriguingly, Cx43 protein levels were low in glia cells in the cerebral cortex where GBMs are frequently found [53], despite that glia cells in basal ganglia and hippocampus expressed high levels of Cx43 (Supplemental Fig. S3B). Recent transcriptomic and proteomic analyses in the same high-grade glioma cell lines (DepMap) showed a strong correlation (*r* = 0.6; *P* = 0.03) between mRNA levels and protein levels of Cx43 (Supplemental Fig. S3C) and that levels of Cx43 proteins were higher than those of other connexins (Supplemental Fig. S3D). Collectively, Cx43 mRNA and proteins are expressed at higher levels than other connexins in a subpopulation of GBMs.

**Fig. 2:**
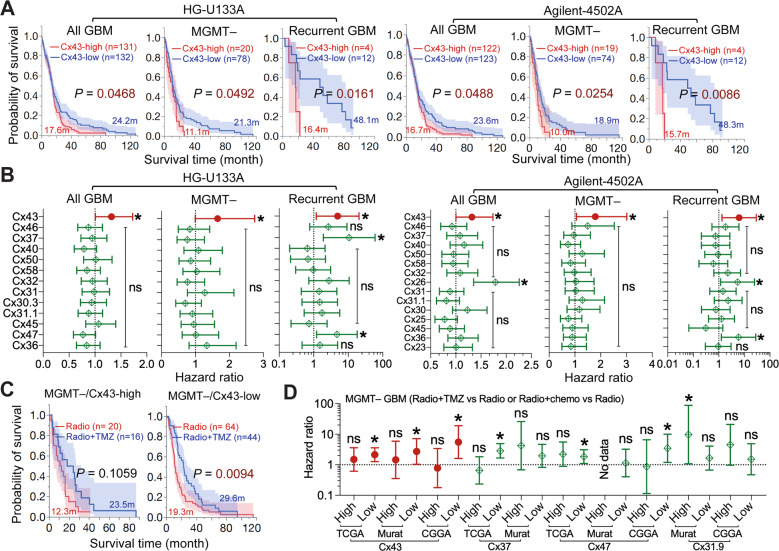
mRNA levels of Cx43, but not other connexins, correlate with GBM poor prognosis and chemoresistance. GBM datasets were retrieved from cBioPortal, GlioVis, or CGGA data portal. Immunostaining results of high-grade glioma were obtained from the Human Protein Atlas. **A** Kaplan–Meier analysis in the TCGA HG-U133A and Agilent-4502A microarray datasets. Patients were divided into Cx43-high (red; top 25 percentile) or Cx43-low (blue; bottom 25 or 75 percentile) based upon Cx43 mRNA levels in all GBM (All GBM), MGMT promoter methylated primary GBM (MGMT–), or recurrent GBM only (Recurrent GBM). Case number (n), mean survival time in months (m), and log-rank *P* values are shown. Red or blue shadows represent 95% confidence interval of Cx43-high or Cx43-low group, respectively. **B** Cox univariate analysis in the TCGA HG-U133A and Agilent-4502A microarray datasets. The Cox univariate analysis employs the Cox proportional hazards model to yield a hazard ratio that indicates risk levels of death in patients with high mRNA levels of connexins compared to those with low levels. The resulting *P* value determines significance of hazard ratio. Cx43 is highlighted in red. **C** Kaplan–Meier analysis in TCGA HG-U133A. MGMT– GBMs were divided into Cx43-high or Cx43-low group as described above. Patients treated with radiation alone (Radio; red) were compared to patients treated with both radiation and TMZ (Radio+TMZ; blue). **D** Cox univariate analysis in TCGA HG-U133A, Murat GBM, and CGGA recurrent GBM. MGMT-deficient primary GBMs or recurrent GBMs were divided into Cx43-high or Cx43-low groups. One-Way ANOVA was used to determine statistical significance. *P < 0.05. ****P < 0.0001. ns: not significant. Error bars are standard deviations.

Survival analyses (Fig. 2A) revealed that high levels of Cx43 mRNA were associated with poor prognosis of all GBM patients (All GBM), primary GBMs with promoter methylation of MGMT (MGMT–) [6], and recurrent GBMs (recurrent GBM). These results were consistent with our previous studies [21] and the finding that recurrent GBMs are often refractory to TMZ [5]. In contrast to these results, no correlation was found between Cx43 mRNA levels and the prognosis of primary or MGMT-expressing (MGMT + ) GBMs (Supplemental Fig. S4A). Cox univariate analyses, which yield a hazard ratio (HR) that determines chance of death (HR > 1 indicates high risk of death), showed that Cx43-high patients displayed high risk of death in the group of All GBM, MGMT–, and Recurrent GBM (Fig. 2B), but not in the group of primary or MGMT + GBM (Supplemental Fig. S4B). Other connexins, however, had variable HRs exhibiting either no statistical significance or not consistently significant in all three groups. Similar results were found in Murat GBM (Supplemental Fig. S5) and recurrent GBMs from CGGA (Supplemental Fig. S6). Our results demonstrate that mRNA levels of Cx43, but not other connexins, correlate with the prognosis of MGMT– or recurrent GBMs.

TMZ improves GBM prognosis when used in combination with radiation [6]. However, the combination of radiation and TMZ (Radio+TMZ) or chemo (Radio+chemo) showed no difference in the survival of Cx43-high/MGMT– GBMs (Fig. 2C, D and Supplemental Fig. S7), whereas the addition of TMZ or chemo increased the survival of MGMT– GBMs expressing low levels of Cx43 mRNA (Cx43-low). Of note, high levels of Cx37, Cx47, or Cx31.9 did not show similar results (Fig. 2D). In line with our prior studies [21, 41], it is suggested that GBM patients with high levels of Cx43 mRNA are resistant to chemotherapies.

### Cx43 confers resistance to TMZ by activating PI3K

**Fig. 3:**
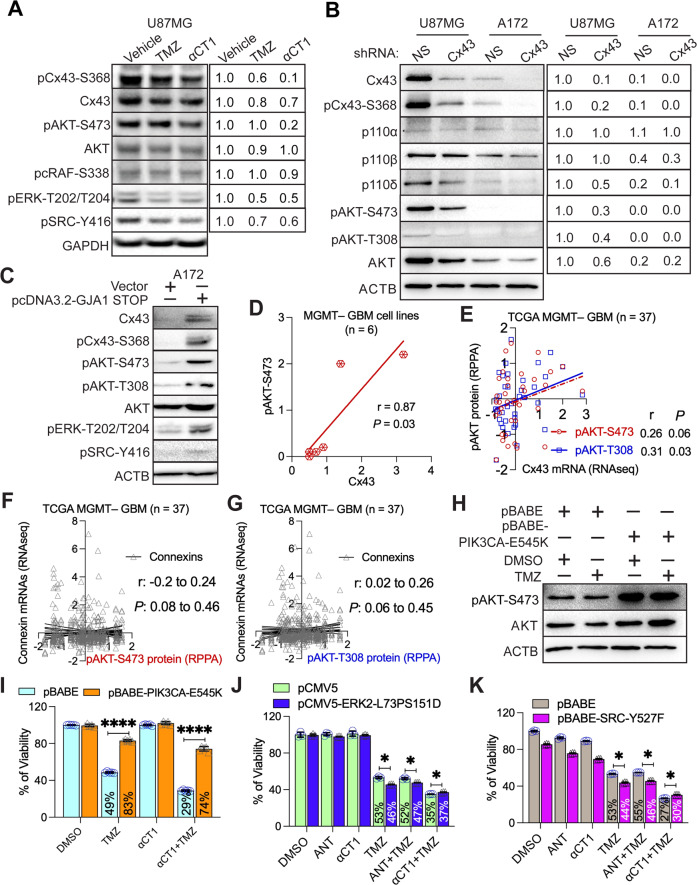
Cx43 blockade inactivates PI3K. **A** Signaling pathways affected by αCT1. Cx43-high U87MG cells were treated with 100 μM αCT1 or 50 μM TMZ for 4 days. pAKT-S473, pcRAF-S338, pERK-T202/T204, and pSRC-Y416 were analyzed using immunoblotting. Glyceraldehyde 3-phosphate dehydrogenase (GAPDH) was the loading control. Band intensities (mean gray value × area) were quantified using Image J. The vehicle DMSO or water was set as 1.0 and each treatment was normalized to the vehicle. **B** PI3K signaling upon depletion of Cx43. U87MG and Cx43-low A172 cells were treated with a non-silencing short hairpin RNA (NS shRNA) or a Cx43 shRNA. Band intensities were quantified using Image J. U87MG cells treated with NS shRNA were set as 1.0. β-actin (ACTB) was the loading control. **C** PI3K signaling in Cx43-overexpressing cells. U87MG cells were transfected with either vector or pcDNA3.2-GJA1 STOP. PI3K, ERK, and SRC signaling was analyzed using immunoblotting. Pearson coefficient correlation analysis between protein levels of Cx43 and pAKT-S473 was performed using Prism 9 in 6 MGMT– GBM cell lines (**D**), mRNA levels of Cx43 and protein levels of pAKT-S473 or pAKT-T308 in MGMT– patients (**E**), or mRNA levels of connexins and protein levels of pAKT-S473 (**F**) and pAKT-T308 (**G**) in MGMT– GBMs. The Pearson correlation coefficient (*r*) and *P* value that determines statistical significance of the coefficient are shown. Cell line data were retrieved from our previous studies [21, 27]. **H** Expression of PIK3CA-E545K (an active PI3K mutant). U87MG cells were transfected with pBABE or pBABE-PIK3CA-E545K encoding PIK3CA-E545K followed by the treatment of 100 μM TMZ. DMSO is the vehicle control. **I** The effect of PIK3CA-E545K on the αCT1/TMZ-induced growth inhibition. The above-transfected cells were treated with a combination of 100 μM αCT1 and/or 100 μM TMZ for 6 days. Cell viability was measured using the MTS viability assay. Percentages of viability were obtained by normalizing the MTS readings of treatment groups to that of DMSO. **J** The effect of ERK2-L73PS151D on the αCT1/TMZ-induced growth inhibition. U87MG cells were transfected with pCMV5 or pCMV5-ERK2-L73PS151D (encoding an active ERK2 mutant) followed by the treatment of αCT1 or antennapedia peptide (ANT; the control peptide for αCT1) and/or TMZ. **K** The effect of SRC-Y527F on the αCT1/TMZ-induced growth inhibition. U87MG cells were transfected with pBABE or pBABE-SRC-Y527F (encoding an active SRC mutant) followed by the treatment of αCT1 or ANT and/or TMZ. RNA sequencing (RNAseq) data and results of reverse phase protein array (RPPA) were retrieved from the TCGA database. Student’s *t* test was used to determine statistical significance. *P < 0.05; ****P < 0.0001. Three independent experiments were performed to yield standard deviations (error bars).

Next, we explored how Cx43 confers TMZ resistance. We have previously shown that the Cx43 peptide inhibitor αCT1 inactivates PI3K [21], leading us to hypothesize that Cx43 activates PI3K to induce TMZ resistance. To test this hypothesis, we treated Cx43-high/TMZ-resistant U87MG cells with TMZ or αCT1. αCT1 blocked phosphorylation of Cx43 at serine 368 (Fig. 3A, pCx43-S368), an indicator of Cx43 activity [54]. As expected, αCT1 significantly decreased the phosphorylated form of AKT serine/threonine kinase (pAKT-S473**;** Fig. 3A and Supplemental Fig. S8A). Previous research [55, 56] has suggested that Cx43 regulates the activity of the mitogen-activated protein kinase (MAPK) pathway, including the RAF proto-oncogene serine/threonine-protein kinase (RAF)/extracellular-signal-regulated kinase (ERK) cascade and the SRC proto-oncogene non-receptor tyrosine kinase (SRC) pathway. αCT1 modestly reduced levels of pcRAF-S338, pERK-T202/T204, or pSRC-Y416 (Fig. 3A). To corroborate results from αCT1, we knocked down Cx43 using an shRNA. Cx43 shRNA decreased levels of Cx43 and pCx43-S368 and inactivated PI3K/AKT in U87MG cells, but not in Cx43-low A172 cells having low levels of pAKTs (Fig. 3B and Supplemental Fig. S8B). Moreover, ectopically expressing Cx43 in A172 cells activated PI3K/AKT and ERK (Fig. 3C). These results have demonstrated that Cx43 regulates PI3K/AKT activity in GBM. Through reanalyzing data from our previous work [21, 27], we detected a strong correlation between Cx43 and pAKT-S473 in six MGMT– GBM cell lines (Fig. 3D and Supplemental Table S2). A positive trend was also found between levels of Cx43 mRNA and pAKT-S473 or pAKT-T308 in 37 MGMT– GBM patients in the TCGA dataset (Fig. 3E). Other connexins, however, failed to show statistically significant correlations with either pAKT-S473 (Fig. 3F) or pAKT-T308 (Fig. 3G).

To determine whether PI3K is required for αCT1-medicated chemo-sensitization, we overexpressed PIK3CA-E545K, a PI3K mutant that constitutively activates PI3K, in U87MG cells (Fig. 3H). PIK3CA-E545K counterbalanced the growth inhibition induced by TMZ or by a combination of TMZ and αCT1 (Fig. 3I). This counteraction was not seen in U87MG cells expressing an active mutant of ERK (ERK2-L73PS151D; Fig. 3J) or SRC (SRC-Y527F; Fig. 3K). These results suggest that the inhibition of PI3K is important for αCT1 to restore TMZ sensitivity.

### αCT1 inactivates PI3K independent of Cx43-channels and Cx43 selectively binds to a PI3K catalytic subunit

**Fig. 4:**
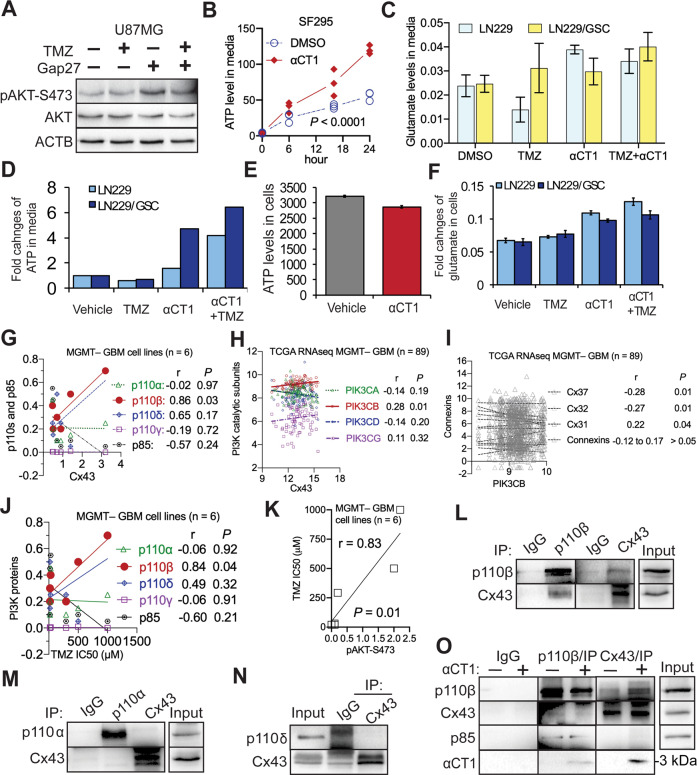
αCT1 inactivates PI3K independent of Cx43-channels and Cx43 selectively binds to p110β. **A** The effect of Gap27 on PI3K signaling. U87MG cells were treated with 100 μM TMZ or 100 μM Gap27. PI3K signaling was assessed by immunoblotting. **B** ATP release from Cx43-high/MGMT–/TMZ-resistant SF295 cells. Cells were treated with 100 μM αCT1. Culture media were collected at different time points. ATP was measured using a colorimetric assay as described in Methods. One-way ANOVA was used to determine statistical significance. **C** Glutamate release in Cx43-low/MGMT–/TMZ-sensitive LN229 or Cx43-high/MGMT–/TMZ-resistant LN229/GSC cells. Cells were treated with 100 μM TMZ and/or 100 μM αCT1. Glutamate in culture media was determined using a colorimetric assay. **D** ATP release in LN229 and LN229/GSC cells. **E** ATP levels inside of SF295 cells. **F** Glutamate levels inside of LN229 and LN229/GSC cells. GSC glioblastoma stem cells. Pearson coefficient Correlation between protein levels of Cx43 and PI3K catalytic subunits in 6 MGMT– GBM cell lines (**G**), mRNA levels of Cx43 and PI3K catalytic subunits in MGMT– GBM patients (**H**), mRNA levels of PIK3CB and connexins in MGMT– GBM patients (**I**), protein levels of p110 proteins and IC50s of TMZ in 6 MGMT– GBM cell lines (**J**), or protein levels of pAKT-S473 and IC50s of TMZ in 6 MGMT– GBM cell lines (**K**). Cell line data were retrieved from our previous studies [21, 27]. RNAseq data were retrieved from the TCGA database. The Pearson correlation coefficient r and corresponding *P* are shown. Co-immunoprecipitation of Cx43 and p110β (**L**), p110α (**M**), or p110δ (**N**) was performed in U87MG cells as described in Methods. **O** Co-immunoprecipitation of Cx43 and p110β in U87MG cell lysates treated with 100 μM αCT1. αCT1 is about 3 kDa and recognized by the Cx43 antibody. IP immunoprecipitation. Rabbit IgG was used as the control. Three independent experiments were performed to yield standard deviations (error bars).

Because the Cx43-CT regulates the activity of Cx43-channels [57], it is possible that small molecules such as ATP or glutamate released from Cx43-channels activate PI3K in GBM cells as they do in astrocytes [58]. To test this possibility, we treated U87MG cells with Gap27, a Cx43 peptide inhibitor that targets the second extracellular loop of Cx43 and blocks Cx43-channels [59]. Gap27, however, slightly increased AKT activity (Fig. 4A). Next, we examined the levels of ATP or glutamate in Cx43-high SF295 and LN229/GSC as well as Cx43-low LN229 cells. Regardless of levels of Cx43, levels of ATP or glutamate in culture media either elevated or remained unchanged in αCT1-treated cells (Fig. 4B–D). This could be explained by the enhanced permeability of Cx43 hemichannels upon dephosphorylation of Cx43 by αCT1 (Fig. 3A) [60]. ATP or glutamate levels remained unchanged in cells (Fig. 4E, F). Our results suggest that Cx43-channels are dispensable for PI3K activation in GBM cells.

Cx43-CT interacts with certain signaling molecules [55]. It is likely that Cx43 binds to PI3K catalytic subunits to activate PI3K. The Class I PI3K family consists of four highly homologous catalytic subunits: PI3K catalytic subunits α, β, δ, and γ (PIK3CA, PIK3CB, PIK3CD, and PIK3CG) encoding p110α, p110β, p110δ, and p110γ, respectively [61]. Our previous work has demonstrated that PI3K catalytic subunits play divergent roles in GBM cell survival, with p110β being the most dominant isoform in GBM [27]. To determine whether PI3K catalytic subunits also function divergently in Cx43-induced PI3K activation, we reanalyzed protein expression data in six MGMT– GBM cell lines (Supplemental Table S3). Levels of Cx43 protein showed a positive and statistically significant correlation with those of p110β, but not other p110s or the regulatory subunit p85 (Fig. 4G). mRNA levels of Cx43 also positively corresponded with those of PIK3CB, but not other PI3K subunits, in 89 MGMT– GBM patients (Fig. 4H). In the same GBM patients, PIK3CB displayed no or negative correlation with other connexins, except Cx31 (Fig. 4I). Such a positive correlation between Cx43 mRNA and PIK3CB mRNA was recapitulated in multiple GBM datasets (Supplement Fig. S9) and further verified by the finding that GBMs with high levels of pAKT-S473 or p110β, but not other p110s, were resistant to TMZ indicated by increased TMZ IC50s (Fig. 4J, K). To further probe the molecular details of Cx43-induced PI3K activation, we monitored protein-protein interactions between Cx43 and p110 proteins. Cx43 was co-precipitated with p110β (Fig. 4L) but not with p110α or p110δ (Fig. 4M, N), demonstrating a selective binding between Cx43 and p110β. We did not examine p110γ because this subunit is not detectable in GBM [27]. To determine whether αCT1 binds to Cx43 and/or p110β, we treated U87MG cell lysates with αCT1 and found that αCT1 was pulled down together with p110β and Cx43 (Fig. 4O). In the presence of αCT1, more p110β was found in the Cx43 precipitate. This might be because the Cx43 antibody is able to precipitate αCT1- and Cx43-bound protein complexes. Taken together, αCT1 inactivates PI3K independent of Cx43-channels and Cx43 selectively binds to p110β.

### A combination of αCT1 and p110β-selective inhibitors overcomes TMZ resistance

αCT1 alone increases the sensitivity of LN229/GSC xenograft tumors to TMZ [21]; however, the short half-life of αCT1 demands high concentrations and repeated drug delivery, which may limit its therapeutic potential. Prompted by the above results, we tested the combination of αCT1 and p110β-selective inhibitors in cultured cells and in mice. To achieve a synergistic therapeutic effect of multiple drugs, we optimized the dose of each individual drug in U87MG cells. By varying doses of TMZ or a p110β-selective inhibitor TGX-221 (TGX), we found that the double combination of 50 μM TMZ and 20 μM TGX-221 only reduced the viability of U87MG cells by ~50% (Supplemental Fig. S10A, B). However, the addition of αCT1 as low as 10 μM greatly increased the cytotoxic effect of the TMZ/TGX-221 double combination (Supplemental Fig. S10C). Next, we assessed synergistic drug effects using the Bliss independent model, a method commonly used to measure drug synergy [39, 40]. This model yields Excess Over Bliss (EOB) scores. EOB > 0% indicates a synergistic effect; EOB = 0% means an additive drug effect; EOB < 0% refers to an antagonistic effect. 2.5–10 μM αCT1 together with TMZ/TGX-221 only yielded a weak synergistic effect on cell viability, whereas 12.5 to 50 μM αCT1 exhibited a much stronger synergistic inhibition on cell viability (Supplemental Fig. S10D).

**Fig. 5:**
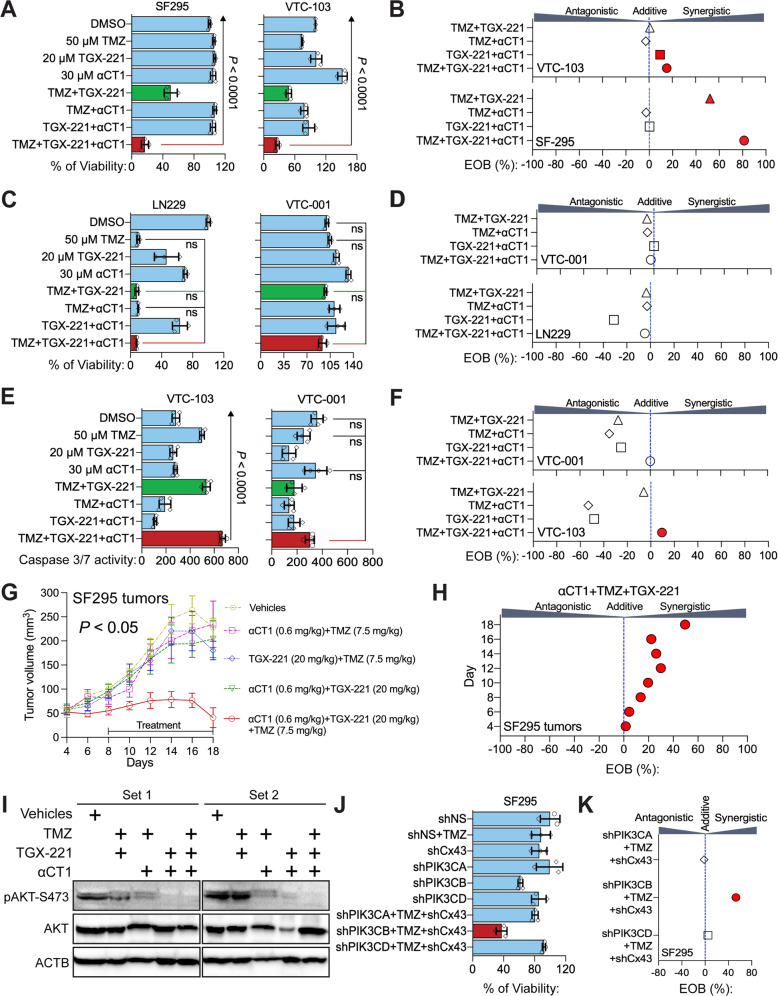
A combination of αCT1 and TGX-221 overcomes TMZ resistance in vitro and in vivo. **A** The effect of the αCT1/TGX/TMZ combo in Cx43/p110β-high/MGMT–/TMZ-resistant SF295 and VTC-103 cells. Cells were treated with 50 μM TMZ, 20 μM TGX-221, and/or 30 μM αCT1 including single agents, double combinations, and the αCT1/TGX/TMZ combo. This scheme has been repeated in experiments presented hereafter. Cell viability was determined using the MTS viability assay. Percentage of cell viability was obtained by normalizing the MTS reading of treatment groups to that of the DMSO group. **B** EOB scores were calculated using the Bliss Independence model. The drug combination is synergistic if EOB is more than 0%, additive if EOB equals to 0%, or antagonistic if EOB is less than 0%. **C** The effect of the αCT1/TGX/TMZ in Cx43/p110β-low/MGMT–/TMZ-sensitive LN229 and TMZ-resistant VTC-001 cells. **D** EOB scores of drug combinations in LN229 and VTC-001 cells. **E** Caspase 3/7 activity in VTC-103 and VTC-001 cells. The activity of cleaved caspase 3/7 (active) was determined using a luminescence assay as described in Methods. Shown are luminescence readings. **F** EOB scores of drug combinations in VTC-103 and VTC-001 cells. **G** The effect of αCT1/TGX/TMZ combo on SF295 xenograft tumors. SF295 cells were subcutaneously injected into immuno-deficient mice. 8 days later, mice were treated with TMZ, TGX-221, or αCT1 through intraperitoneal or intratumoral injection every other day until day 18. Tumor volumes are shown. **H** EOB scores of drug combinations in SF295 tumors at different days. **I** Immunoblotting of PI3K signaling in SF-295 tumors. Proteins were directly extracted from homogenized tumor tissues and analyzed using western-blotting as described in Methods. ACTB is the loading control. Two repeated experiments are shown. **J** The effect of shRNA of Cx43 or PI3K catalytic subunits on the TMZ sensitivity of SF295 cells. Cells were transfected with NS shRNA or shRNA of Cx43, PIK3CA, PIK3CB, or PIK3CD followed by the treatment of 50 μM TMZ. Cell viability was determined using the MTS viability assay. Percentages of cell viability were obtained by normalizing the MTS readings of treatment groups to that of shNS group. **K** EOB scores of drug combinations in SF295 cells. One-way ANOVA with Dunnett test for correcting multiple comparisons or Student’s *t* test was used to determine statistical significance. *P < 0.05; ns not significant. Drug combinations with strong synergistic effect were marked in red. Three independent experiments were performed to yield standard deviations (error bars).

Based on these results, 30 μM αCT1, 20 μM TGX-221, and 50 μM TMZ were used in a triple combination named αCT1/TGX/TMZ combo. The αCT1/TGX/TMZ combo synergistically reduced the viability of MGMT– /TMZ-resistant SF295, VTC-103, and VTC-003 cells (Fig. 5A and Supplemental Fig. S11A) that express high levels of Cx43 and p110β [21, 27]. Notably, VTC-103, VTC-003, and other VTC lines described hereafter were derived from freshly dissected GBM tumors [21, 27]. EOB scores of the αCT1/TGX/TMZ combo were significantly higher than those of double combinations (Fig. 5B and Supplemental Fig. S11B). This synergistic effect was, however, not found in MGMT–/TMZ-sensitive LN229 and A172 or MGMT–/TMZ-resistant VTC-001 and VTC-005 (Fig. 5C, D and Supplemental Figs. S11C, D and S12) whose levels of Cx43 and p110β are low [21, 27]. The αCT1/TGX/TMZ combo synergistically activated apoptosis in VTC-103 cells (Fig. 5E, F), whereas apoptosis was not synergistically induced in VTC-001 cells. To verify our in vitro studies in vivo, we treated mice bearing SF295 xenograft tumors with 7.5 mg/kg TMZ [21] and 20 mg/kg TGX-221 [27] through intraperitoneal injection in conjunction with 0.6 mg/kg αCT1 (equivalent to 30 μM) through intratumoral injection. The αCT1/TGX/TMZ combo (red line) stopped tumor growth (Fig. 5G, *P* < 0.05), whereas double combinations (green, yellow, purple, or blue line) exhibited limited to no inhibition. While EOB scores of the triple combo increased over time, culminating on day 18 (Fig. 5H), Immunoblotting verified that αCT1 and TGX-221 together significantly decreased AKT phosphorylation (Fig. 5I). This confirmed a strong synergy amongst αCT1, TGX-221, and TMZ in vivo. To verify that the synergistic cytotoxicity is due to the blockade of Cx43/p110β, we knocked down Cx43 and individual PI3K catalytic subunits using shRNAs. Depletion of p110β, but not p110α or p110δ, blocked the growth of SF295 cells (Fig. 5J) and only the combination of PIK3CB shRNA, Cx43 shRNA, and TMZ yielded synergistic inhibition of cell viability (Fig. 5K, red circle).

To corroborate results from TGX-221, we tested another p110β-selective inhibitor GSK2636771 (GSK), which has been used in a clinical study [62]. αCT1/GSK/TMZ combo entailing 25 μM GSK2636771, 30 μM αCT1, and 50 μM TMZ synergistically blocked the viability of VTC-103 cells and U87MG cells, but not the viability of LN229 cells (Supplemental Fig. S13). αCT1/GSK/TMZ has achieved the same synergistic inhibition of GBM cell viability as the αCT1/TGX/TMZ combo. To determine the toxicity of these combinations on normal cells, we treated astrocytes with αCT1/TGX/TMZ or αCT1/GSK/TMZ. These drug combinations did not increase TMZ alone-induced growth inhibition in astrocytes (Supplemental Fig. S14), suggesting that addition of αCT1 and p110β-selective inhibitors does not exacerbate non-selective toxicity of TMZ to the normal brain. Collectively, our results demonstrate that simultaneously targeting Cx43 and p110β diminishes TMZ resistance.

## Discussion

**Fig. 6:**
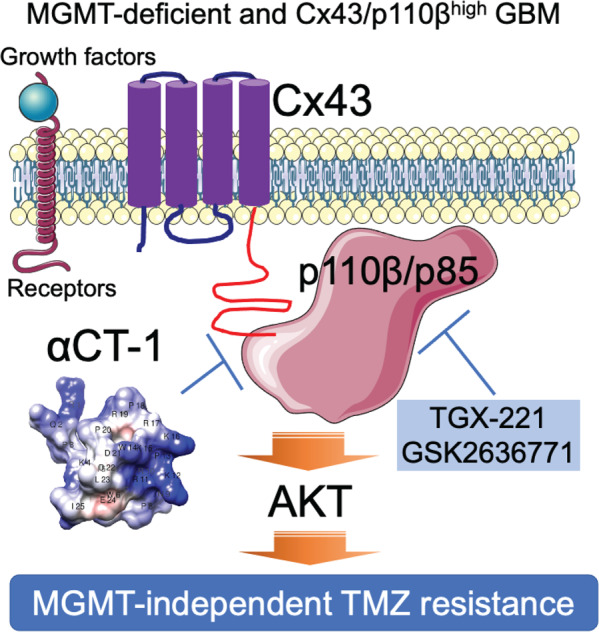
A model illustrates Cx43/PI3K-mediated TMZ resistance independent of MGMT. In MGMT-deficient GBMs, after growth factors bind to their cell surface receptors, Cx43 is expected to recruit p110β/p85 signaling complexes to the membrane through a selective binding to p110β. This in turn activates PI3K/AKT signaling and induces MGMT-independent TMZ resistance. It is also expected that αCT1, a mimetic peptide of Cx43 CT, blocks the interaction between Cx43 and p110β, thereby inactivating p110β and overcoming TMZ resistance. Moreover, p110β-selective inhibitors TGX-221 and GSK2636771, which block p110β kinase activity, synergize with αCT1 to restore TMZ sensitivity in MGMT-deficient GBMs.

In this report, we have identified the molecular details underlying Cx43-induced MGMT-independent TMZ resistance. As illustrated in a model proposed in Fig. 6, Cx43 perhaps binds to p110β/p85 signaling complex upon receiving signals from extracellular cues (i.e., growth factors). This selective binding brings the p110β/p85 signaling complex to the membrane and subsequently activates AKT. Activated PI3K/AKT signaling renders GBM cells resistant to TMZ, which is independent of MGMT. This model not only explains how a gap junction protein regulates chemoresistance through its non-channel functions but also provides a strong rationale for developing combinational therapies to overcome TMZ resistance. Indeed, αCT1 was found in the Cx43/p110β precipitates (Fig. 4O), suggesting that this Cx43-mimetic peptide likely blocks interactions between Cx43 and p110β. It is therefore anticipated that αCT1 (blocking protein–protein interactions) and p110β-selective inhibitors (blocking PI3K kinase activity) synergistically overcome TMZ resistance, which has been verified in vitro and in vivo in this report (Fig. 5).

Prior studies report that Cx43 mRNA and protein are detected in ~20–60% of GBM patients [15, 41, 47, 48]. In light of the fact that 45% of GBM patients express no MGMT [6, 7], there should be 10% (20% × 50%) to 30% (60% × 50%) of patients that are MGMT-deficient and express high levels of Cx43. Congruent with this expectation, we have found that 16.7% of MGMT-deficient GBM patients express high levels of Cx43 [21]. That being said, around 20% of Cx43-high GBM patients may be refractory to TMZ treatment in the clinic. Therefore, the combinational treatment developed herein will benefit these patients, thereby having an important impact on future therapeutic intervention. Previous work has also revealed that, with the exception of Cx43, overexpression or inhibition of Cx30, Cx32, Cx26, or Cx46 also blocks the growth of rat or human glioma cells [63–70]. However, contradictory to these results, other studies show that Cx30 and Cx32 have no effect on glioma growth [66, 71, 72]. In line with the fact that Cx43 levels are much higher than other connexins in GBM (Fig. 1) and the finding that Cx43 controls chemoresistance (Figs. 2–5), Cx43 is therefore the prime therapeutic target for GBM among all connexins.

Cx43 has long been considered as a tumor suppressor for glioma because overexpression of Cx43 leads to remarkable growth inhibition [73] and levels of Cx43 mRNA and protein inversely correlate with the aggressiveness of glioma [41]. However, drawbacks in these studies have made the tumor-suppressive activity of Cx43 questionable. For example, while ectopically expressing Cx43 does inhibit tumor cell growth, it is unclear whether the loss of endogenous Cx43 in normal glial cells promotes gliomagenesis as other tumor suppressors do, namely p53 and NF-1. Nonetheless, it is possible that gap junction intercellular communication controlled by Cx43 is GBM suppressive because the loss of this communication promotes oncogene-induced transformation [74]. In contrast to these studies, we have established a tumor-promoting role of Cx43 in GBM. Cx43, whose mRNA levels are the highest among all connexins, correlates with GBM prognosis and chemoresistance. This membrane channel protein also activates PI3K independent of its channels, selectively binds to p110β, and induces MGMT-independent TMZ resistance. Therefore, it is likely that Cx43 has multifaceted roles in GBM: Cx43-channels inhibit GBM formation, whereas the non-channel activities elicited by Cx43-CT (e.g., PI3K activation) confer chemoresistance during GBM progression.

## Supplementary information

Supplemental Tables and Figures

Supplemental data-whole blot images
