# Supplementary material for: Connexin 43 confers chemoresistance through activating PI3K
Source: Oncogenesis. 2022 Jan 12;11(1):2. doi: 10.1038/s41389-022-00378-7 (PMC8755794; doi:10.1038/s41389-022-00378-7)
Supplement: Supplementary file 1 — Supplemental Tables and Figures [file 41389_2022_378_MOESM1_ESM.pdf]

Supplemental Data-1

Connexin 43 confers chemoresistance through activating PI3K

Kevin J Pridham, Farah Shah, Kasen R Hutchings, Kevin L Sheng, Sujuan Guo, Min Liu, Pratik Kanabur, Samy Lamouille, Gabrielle Lewis, Marc Morales, Jane Jourdan, Christina L Grek, Gautam G Ghatnekar, Robin Varghese, Deborah F Kelly, Robert G Gourdie, and Zhi Sheng

Supplemental Table S1

	Reagent	Company	Catalog#	Reagent	Company	Catalog#
**Chemicals/ Cell Culture**	GSK2636771	AdooQ Bioscience		TGX-221	AdooQ Bioscience	
	Temozolomide	AbMole BioScience		Puromycin	Millipore-Sigma	
	αCT-1	LifeTein	Custom synthesis	Gap27	LifeTein	Custom synthesis
	DMEM	Life Technologies		EquaFETA FBS	Atlas Biologicals	
	Penicillin/ Streptomycin	Gibco		FBS	Peak Serum	
	MCDB-131	Millipore-Sigma		B-27 supplement	Life Technologies	
	FGF-2	PeproTech		EGF	PeproTech	
	L-Glutamine	Gibco		MTS cell viability assay	Promega	
	Caspase-Glo 3/7 assay	Promega		Bradford assay	Bio-Rad Laboratories	
**Antibodies for western blotting**	Anti-phospho-Cx43-S368	Cell Signaling Technology	CST-3511 (1:1000)	Anti-Cx43	Cell Signaling Technology	CST-3512 (1:1000)
	Anti-phospho-AKT-S473	Cell Signaling Technology	CST-4051 (1:1000)	Anti-phospho-AKT-T308	Cell Signaling Technology	CST-4056 (1:1000)
	Anti-AKT	Cell Signaling Technology	CST-4685 (1:1000)	Anti-phospho-cRAF-S338	Cell Signaling Technology	CST-9247 (1:250)
	Anti-phospho-ERK-T202/T204	Cell Signaling Technology	CST-4377 (1:1000)	Anti-phospho-SRC-Y416	Cell Signaling Technology	CST-2101 (1:1000)

	Anti-p110α	Cell Signaling Technology	CST-4249 (1:1000)	Anti-p110β	Cell Signaling Technology	CST-3011 (1:1000)
	Anti-p110δ	Cell Signaling Technology	CST-34050 (1:1000)	Anti-p85	Cell Signaling Technology	CST-4292 (1:1000)
	Anti-β-actin	Millipore-Sigma	MS-A3854 (1:5000)	Anti-GAPDH	Santa Cruz Biotechnology	SC-25778 (1:1000)
**Immuno-precipitation**	Protease inhibitor	Millipore-Sigma		Phosphatase inhibitor	Millipore-Sigma	
	Anti-Cx43	Millipore-Sigma	MS-C6219 (1:50)	Anti-p110α	Cell Signaling Technology	CST-4249 (1:25)
	Anti-p110β	Cell Signaling Technology	CST-3011 (1:25)	Anti-p110δ	Cell Signaling Technology	CST-34050 (1:25)
	Rabbit IgG	Santa Cruz Biotechnology	SC-2027 (1:400)	Protein G Dynabeads	Thermo-Fisher Scientific	
**shRNAs /plasmids**	Cx43 shRNA	Millipore-Sigma	TRCN 0000059773	PIK3CA shRNA	Thermo-Fisher Scientific	RHS4844-101656239
	PIK3CB shRNA	Thermo-Fisher Scientific	RHS4884-10165656350	PIK3D shRNA	Thermo-Fisher Scientific	RHS4884-101655755
	pBABE-puro	Addgene	1764	pBABE-PIK3CA-E545K	Addgene	12525
	pCMV5-ERK2-Q103A	Addgene	40816	pBABE-SRC-Y527F	Addgene	13660
**Animal experiments**	Matrigel Matrix	Corning		Scid/beige mice	Taconic Biosciences	
**Kits**	Kinase-Glo® Luminescent Kinase Assay	Progema		Amplex™ Red Glutamic Acid/Glutamate Oxidase Assay Kit	Thermo-Fisher Scientific	

Supplemental Table S2

Gene symbol	Gene full name	Alias	Abbreviations
**GJA1**	Gap junction protein alpha 1	Connexin 43	**Cx43**
**GJA3**	Gap junction protein alpha 3	Connexin 46	**Cx46**
**GJA4**	Gap junction protein alpha 4	Connexin 37	**Cx37**
**GJA5**	Gap junction protein alpha 5	Connexin 40	**Cx40**
**GJA8**	Gap junction protein alpha 8	Connexin 50	**Cx50**
**GJA9**	Gap junction protein alpha 9	Connexin 58	**Cx58**
**GJA10**	Gap junction protein alpha 10	Connexin 62	**Cx62**
**GJB1**	Gap junction protein beta 1	Connexin 32	**Cx32**
**GJB2**	Gap junction protein beta 2	Connexin 26	**Cx26**
**GJB3**	Gap junction protein beta 3	Connexin 31	**Cx31**
**GJB4**	Gap junction protein beta 4	Connexin 30.3	**Cx30.3**
**GJB5**	Gap junction protein beta 5	Connexin 31.1	**Cx31.1**
**GJB6**	Gap junction protein beta 6	Connexin 30	**Cx30**
**GJB7**	Gap junction protein beta 7	Connexin 25	**Cx25**
**GJC1**	Gap junction protein gamma 1	Connexin 45	**Cx45**
**GJC2**	Gap junction protein gamma 2	Connexin 47	**Cx47**
**GJC3**	Gap junction protein gamma 3	Connexin 30.2	**Cx30.2**
**GJD2**	Gap junction protein delta 2	Connexin 36	**Cx36**
**GJD3**	Gap junction protein delta 3	Connexin 31.9	**Cx31.9**
**GJD4**	Gap junction protein delta 4	Connexin 40.1	**Cx40.1**
**GJE1**	Gap junction protein epsilon 1	Connexin 23	**Cx23**

Supplemental Table S2. Nomenclature of connexins. Information regarding gene symbols and aliases was retrieved from GeneCards (https://www.genecards.org).

Supplemental Table S3

Cell lines	Cx43	pAKT-S473	p110β	MGMT	TMZ IC50 (μM)
SF295	1.4	2.0	0.5	No	500
U87MG	3.2	2.2	0.7	No	1000
A172	0.5	0.1	0.4	No	30
LN229	0.5	0.0	0.2	No	20
SF268	0.7	0.1	0.3	No	20
SNB75	0.9	0.2	0.2	No	293

Supplemental Table S3. Levels of Cx43, pAKT-S473, p110β, MGMT and TMZ IC50. Data were retrieved from our previous publications (21, 27).

Supplemental Fig. S1

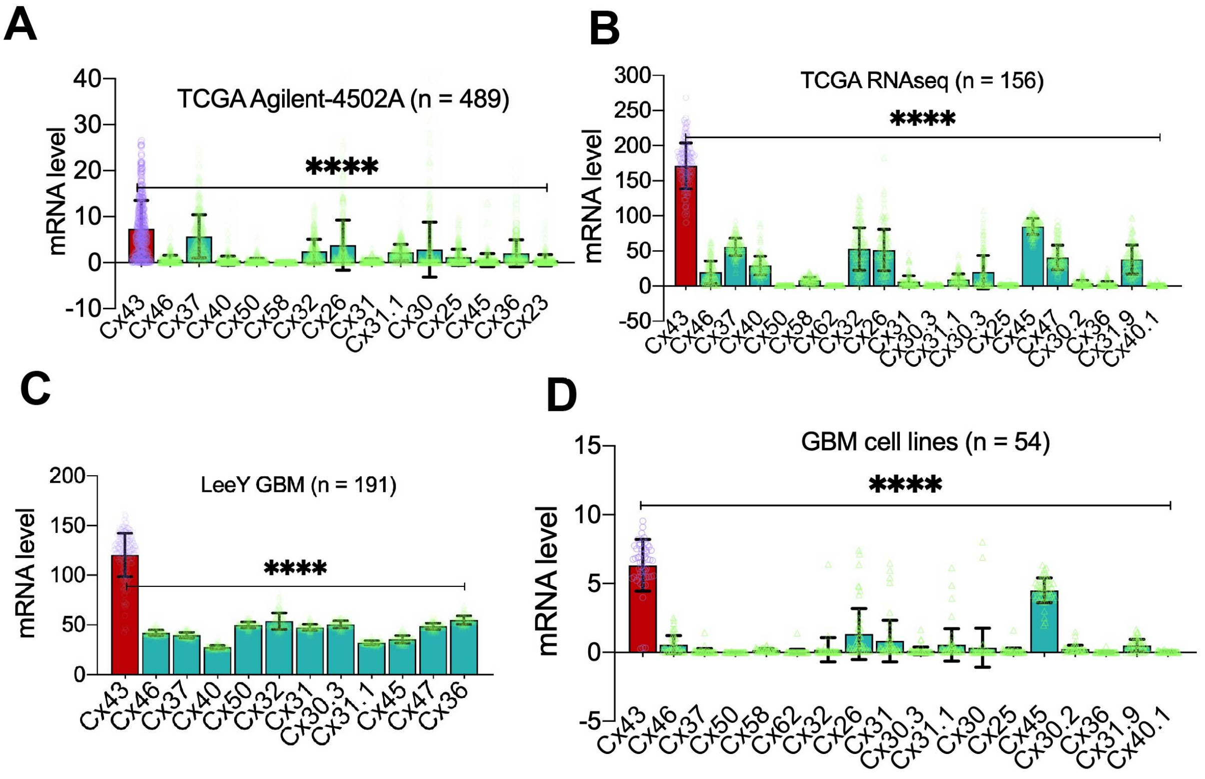

Supplemental Fig. S1. mRNA levels of connexins in GBM. Gene expression data were retrieved from cBioPortal, GlioVis, or DepMap. Shown are mRNA levels of connexins in the TCGA Agilent-4502A microarray (**A**), the TCGA RNAseq (**B**), the LeeY GBM dataset (**C**), and DepMap GBM cell lines (**D**). Case numbers (n) are also shown. Error bars represent standard deviations. Cx43 is highlighted in red and other connexins are in green. Individual data points are also shown (purple for Cx43 and yellow for other connexins). *P* values were obtained using One-Way ANOVA with Dunnett test for correction of multiple comparisons. ****: *P* < 0.0001. All analyses were performed and results were plotted using Prism 9.

Supplemental Fig. S2

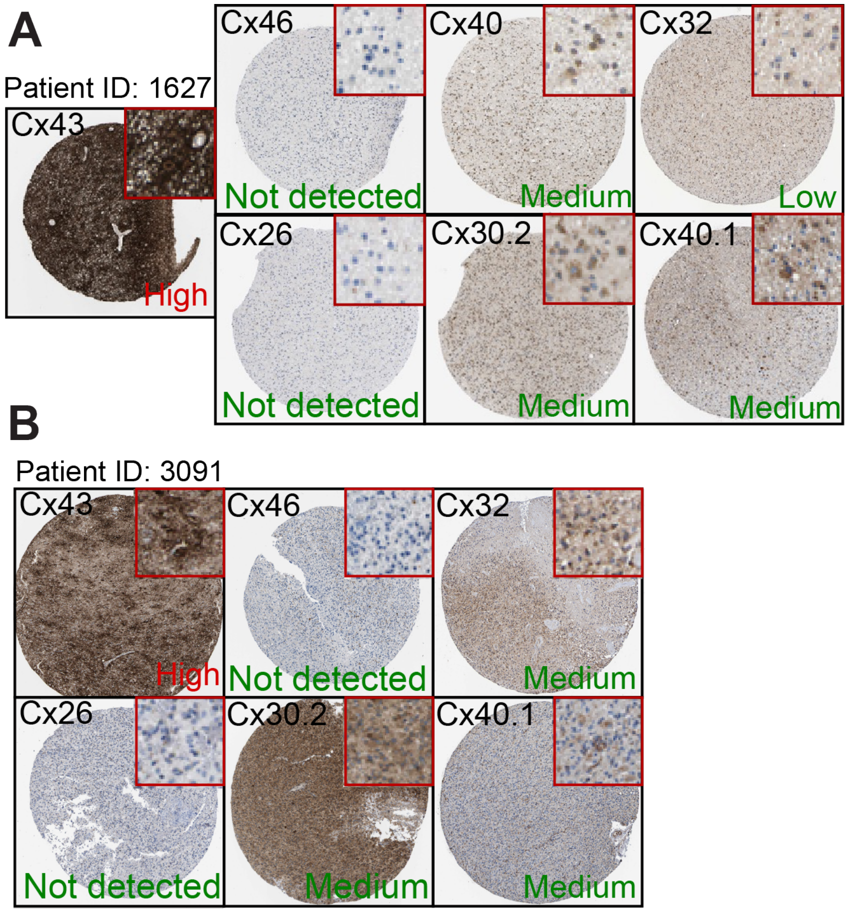

Supplemental Fig. S2. Levels of connexins in high-grade glioma. Immunohistochemical staining images of high-grade glioma were retrieved the Human Protein Atlas. Images of two patient specimens are shown in **A** and **B**, respectively. Inset figures depict details of immunostaining. Levels of staining are highlighted in red (Cx43) or in green (other connexins).

Supplemental Fig. S3

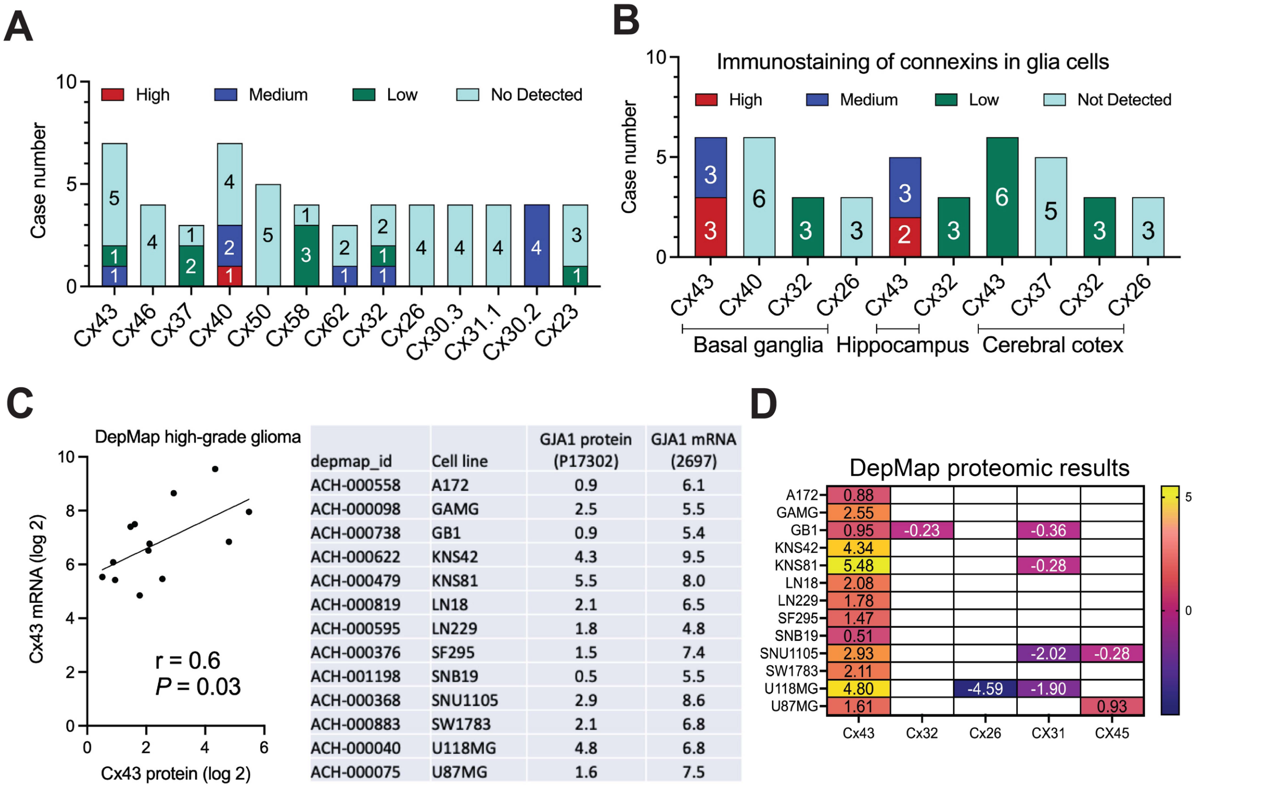

depmap_id	Cell line	GJA1 protein (P17302)	GJA1 mRNA (2697)
ACH-000558	A172	0.9	6.1
ACH-000098	GAMG	2.5	5.5
ACH-000738	GB1	0.9	5.4
ACH-000622	KNS42	4.3	9.5
ACH-000479	KNS81	5.5	8.0
ACH-000819	LN18	2.1	6.5
ACH-000595	LN229	1.8	4.8
ACH-000376	SF295	1.5	7.4
ACH-001198	SNB19	0.5	5.5
ACH-000368	SNU1105	2.9	8.6
ACH-000883	SW1783	2.1	6.8
ACH-000040	U118MG	4.8	6.8
ACH-000075	U87MG	1.6	7.5

Supplemental Fig. S3. Expression of connixins in normal and malignant brain cells and tissues. (**A**) Levels of connexins in low-grade glioma. Case numbers with different immunostaining intensities are shown. Data were retrieved from The Human Protein Atlas. (**B**) Immunostaining results of connexin proteins in glia cells in basal ganglia, hippocampus, and cerebral cortex. (**C**) Correlation of levels between Cx43 mRNA and protein in high-grade glioma cell lines. Proteomic and transcriptomic results in the same cell lines (GBM and astrocytoma) were plotted and Pearson correlation coefficient r was determined using Prism software. Data were retrieved from DepMap. (**D**) DepMap proteomic results of connexin proteins in high-grade glioma cell lines. All analyses were performed and results were plotted using Prism 9.

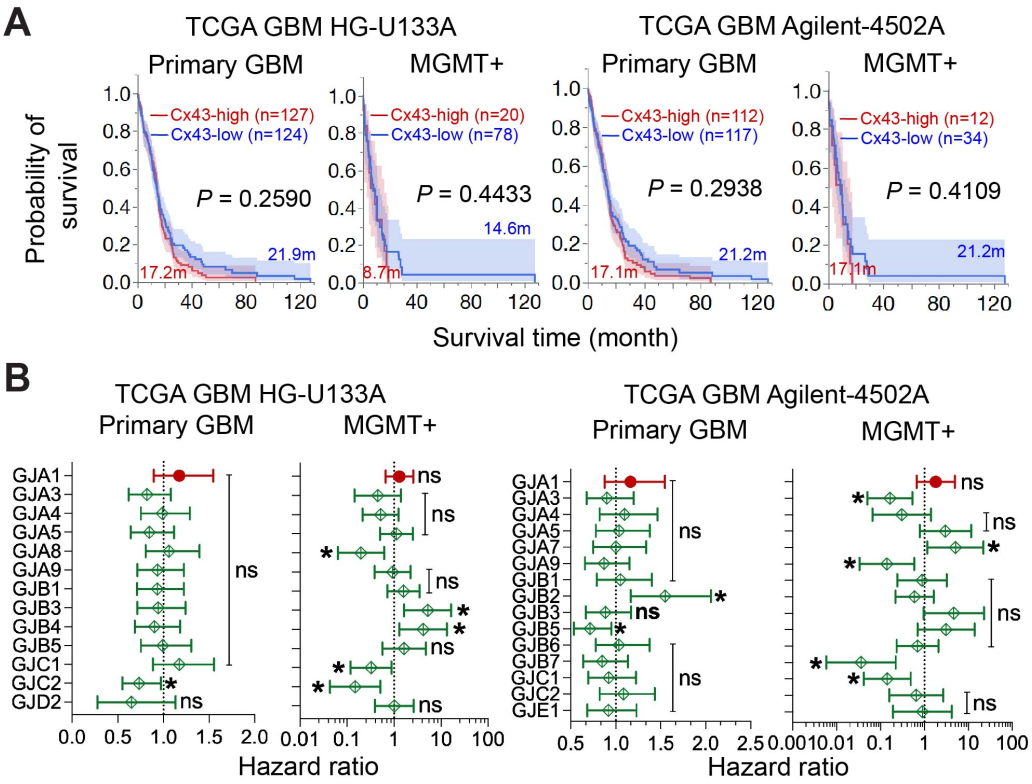

Supplemental Fig. S4. Kaplan-Meier analysis and Cox univariate analysis in the primary or MGMT+ TCGA datasets. Data were retrieved from cBioportal. Patients were divided into Cx43-high (red, top 25 percentile) and Cx43-low (blue, bottom 25 or 75 percentile) based upon Cx43 mRNA levels in primary GBM (Primary GBM) or MGMT-expressing primary GBM (MGMT+). Kaplan-Meier analysis (**A**) and Cox univariate analysis (**B**) were preformed using JMP Pro and results were plotted using either JMP Pro or Prism 9. Case number (n), average survival time in months (m), 95% CI (shadow), long-rank *P* values, and hazard ratios are shown. *: $P < 0.05$. ns: not significant.

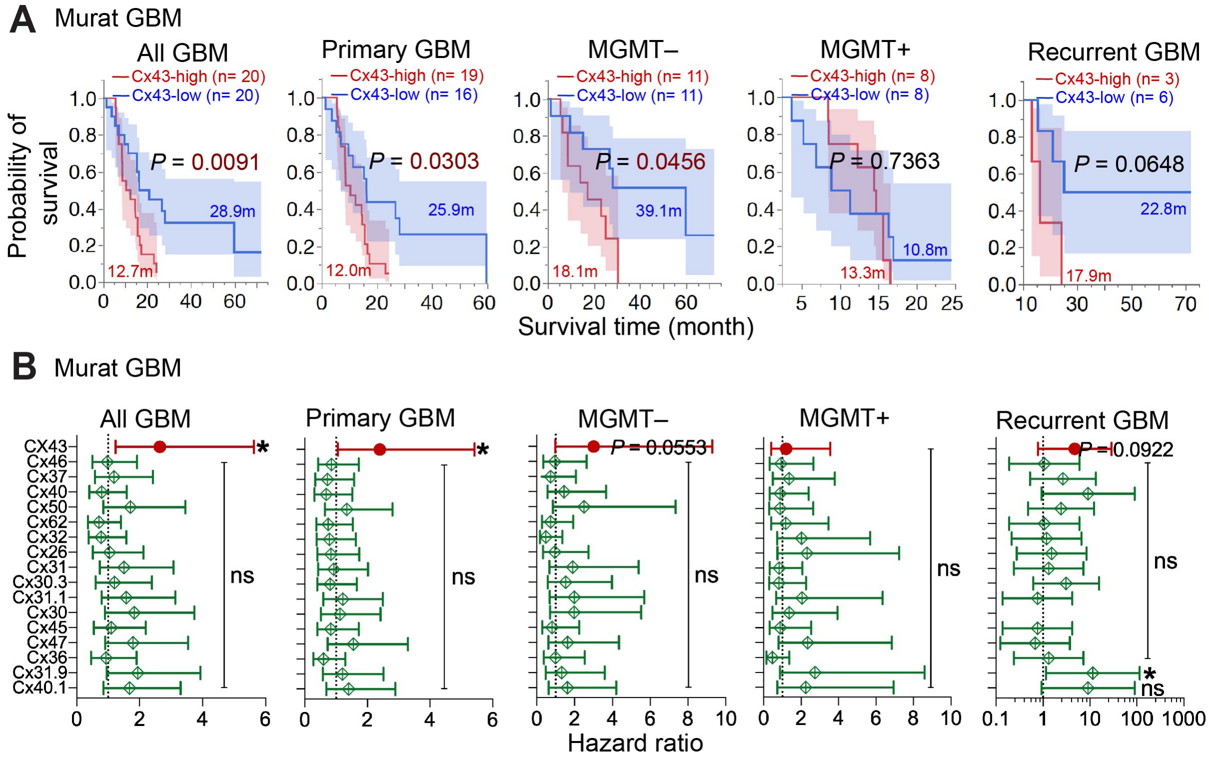

Supplemental Fig. S5. Kaplan-Meier analysis and Cox univariate analysis in the Murat GBM dataset. Data were retrieved from GlioVis. Patients were divided into Cx43-high (red, top 25 percentile) and Cx43-low (blue, bottom 25 or 75 percentile) based upon Cx43 mRNA levels in all GBM (All GBM), primary GBM (Primary GBM), MGMT-deficient primary GBM (MGMT–), MGMT-expressing (MGMT+), or recurrent GBM (Recurrent GBM). Kaplan-Meier analysis (**A**) and Cox univariate analysis (**B**) were performed using JMP Pro and results were plotted using JMP Pro or Prism 9. Case number (n), average survival time in months (m), 95% CI (shadow), long-rank *P* values, and hazard ratios are shown. *: $P < 0.05$. ns: not significant.

Supplemental Fig. S6

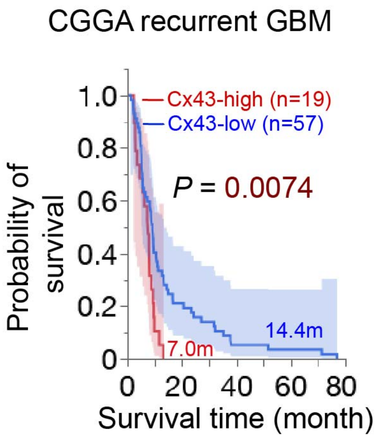

Supplemental Fig. S6. Kaplan-Meier analysis of CGGA recurrent GBMs. Data regarding 76 recurrent GBM were retrieved from the CGGA data portal. Patients were divided into Cx43-hgih (top 25 percentile) or Cx43-low (bottom 75 percentile) based on Cx43 mRNA levels in 76 recurrent GBMs. Kaplan-Meier survival analyses were performed using JMP Pro. Case number (n), average survival time in months (m), and long-rank *P* values are shown.

Supplemental Fig. S7

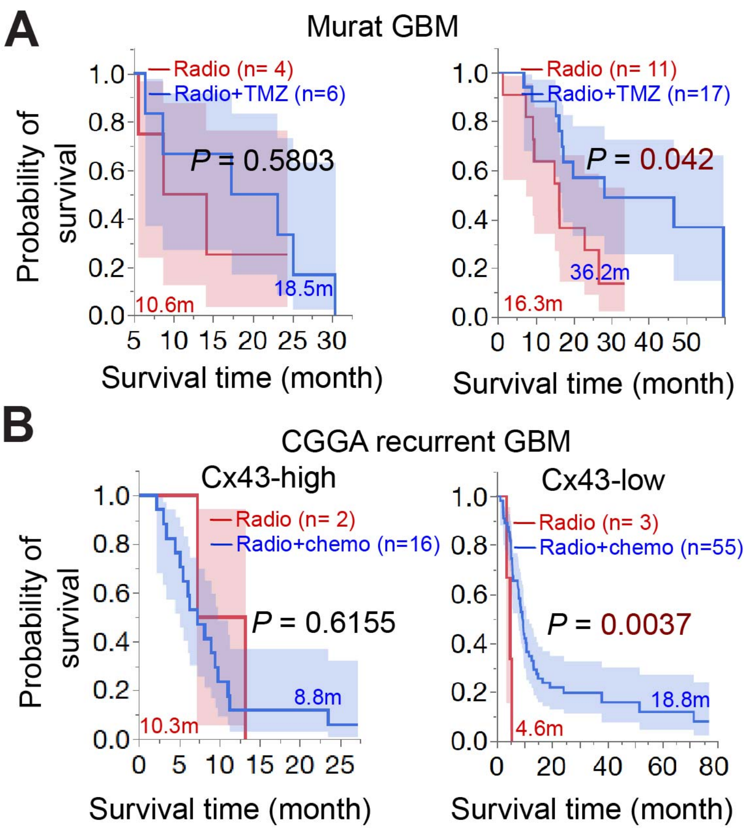

Supplemental Fig. S7. Kaplan-Meier analysis in Murat GBMs and CGGA recurrent GBMs. (**A**) Kaplan Meier analysis in Murat GBM. MGMT– GBMs were divided into Cx43-high (top 25 percentile) or Cx43-low group (bottom 75 percentile). Patients treated with radiation alone (Radio; red) were compared to patients treated with both radiation and TMZ (Radio+TMZ; blue). (**B**) Kaplan Meier analysis in CGGA recurrent GBM. Data regarding 76 recurrent GBM were retrieved from the CGGA data portal. Patients were divided into Cx43-hgih (top 25 percentile) or Cx43-low (bottom 75 percentile) based on Cx43 mRNA levels in 76 recurrent GBMs. Cx43-hgih (top 25 percentile) or Cx43-low (bottom 75 percentile) patients were divided into Radio (red, treated with radiation only) or Radio+chemo (blue, treated with radiation and chemotherapy) based on Cx43 mRNA levels in recurrent GBMs. Kaplan-Meier survival analyses were performed using JMP Pro. Case number (n), average survival time in months (m), and long-rank *P* values are shown.

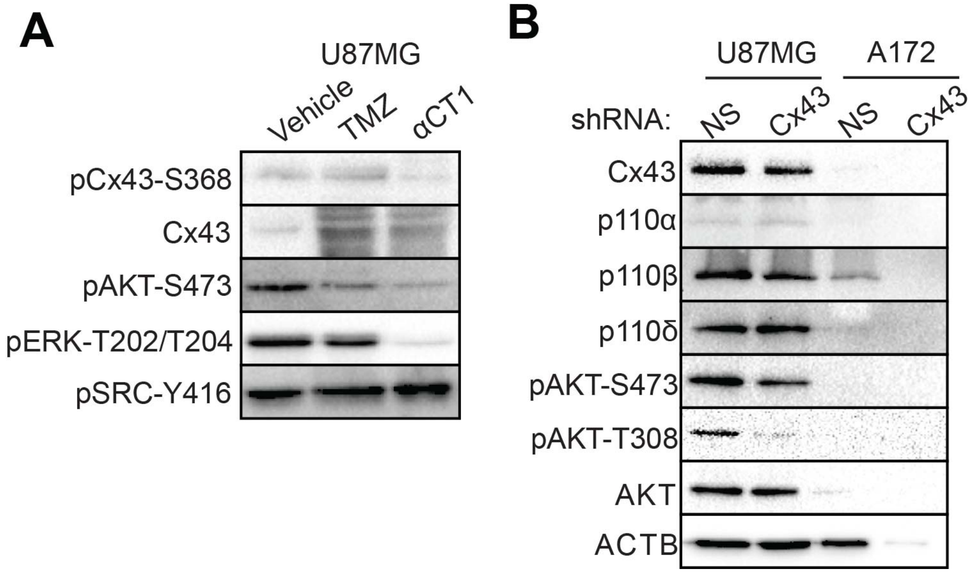

Supplemental Fig. S8. Repeats of αCT1/TMZ treatment and Cx43 knockdown. (**A**) Signaling pathways affected by αCT1. Cx43-high U87MG cells were treated with 100 μM αCT1 or 50 μM TMZ for 4 days. (**B**) PI3K signaling upon depletion of Cx43. U87MG and A172 cells were transiently transfected with viruses containing an NS shRNA or a Cx43 shRNA for 3 days. Signaling molecules were analyzed using immunoblotting.

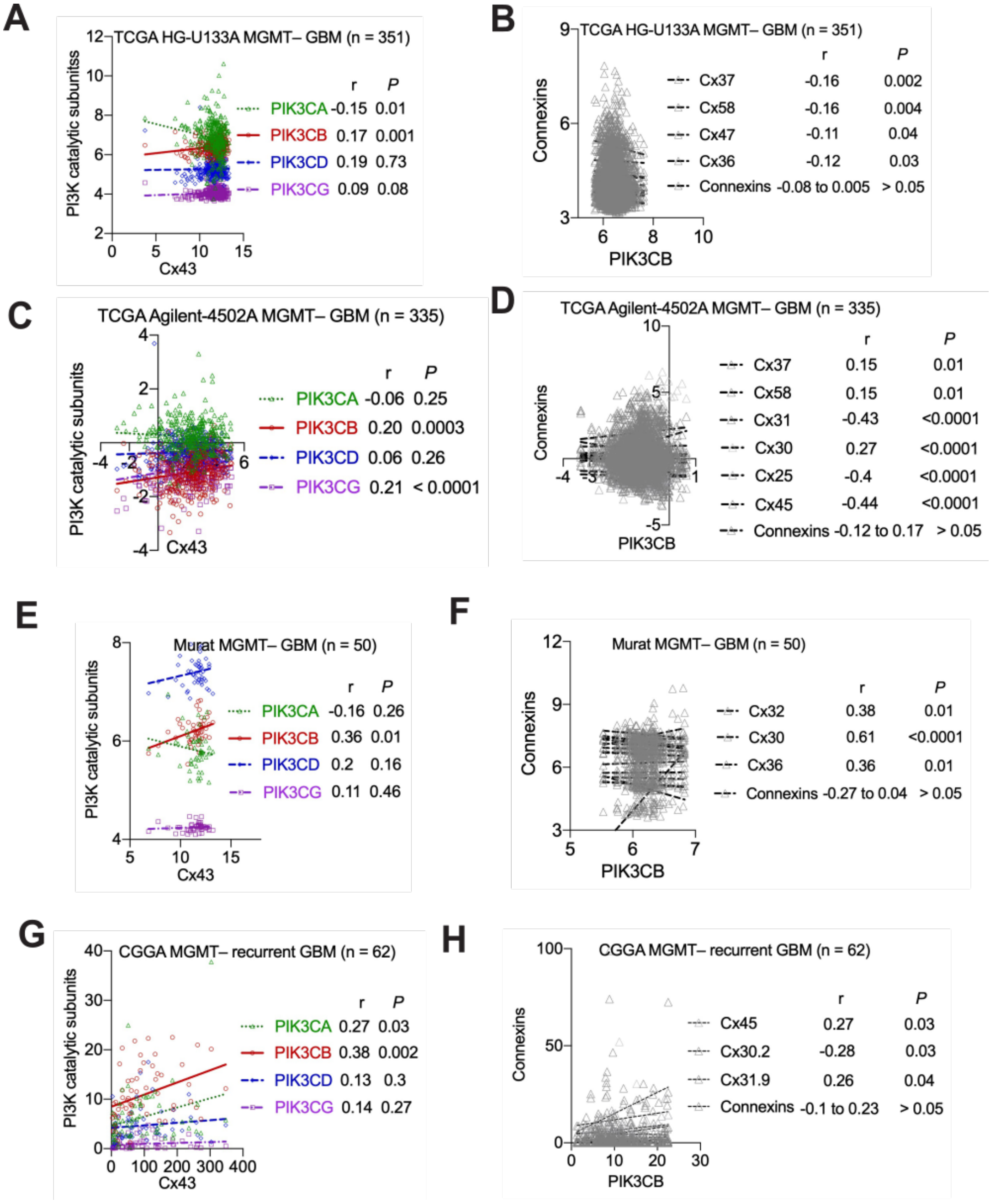

Supplemental Fig. S9. Correlation between connexins and PI3K catalytic subunits. Pearson correlation coefficient assay was performed in different gene expression datasets using Prism 9. mRNA levels of Cx43 were compared to mRNA levels of PI3K catalytic subunits (**A**, **C**, **E**, and **G**) in four different datasets as indicated. mRNA levels of PIK3CB were compared to those of connexin mRNAs (**B**, **D**, **F**, and **H**). The coefficient r and corresponding *P* values are shown.

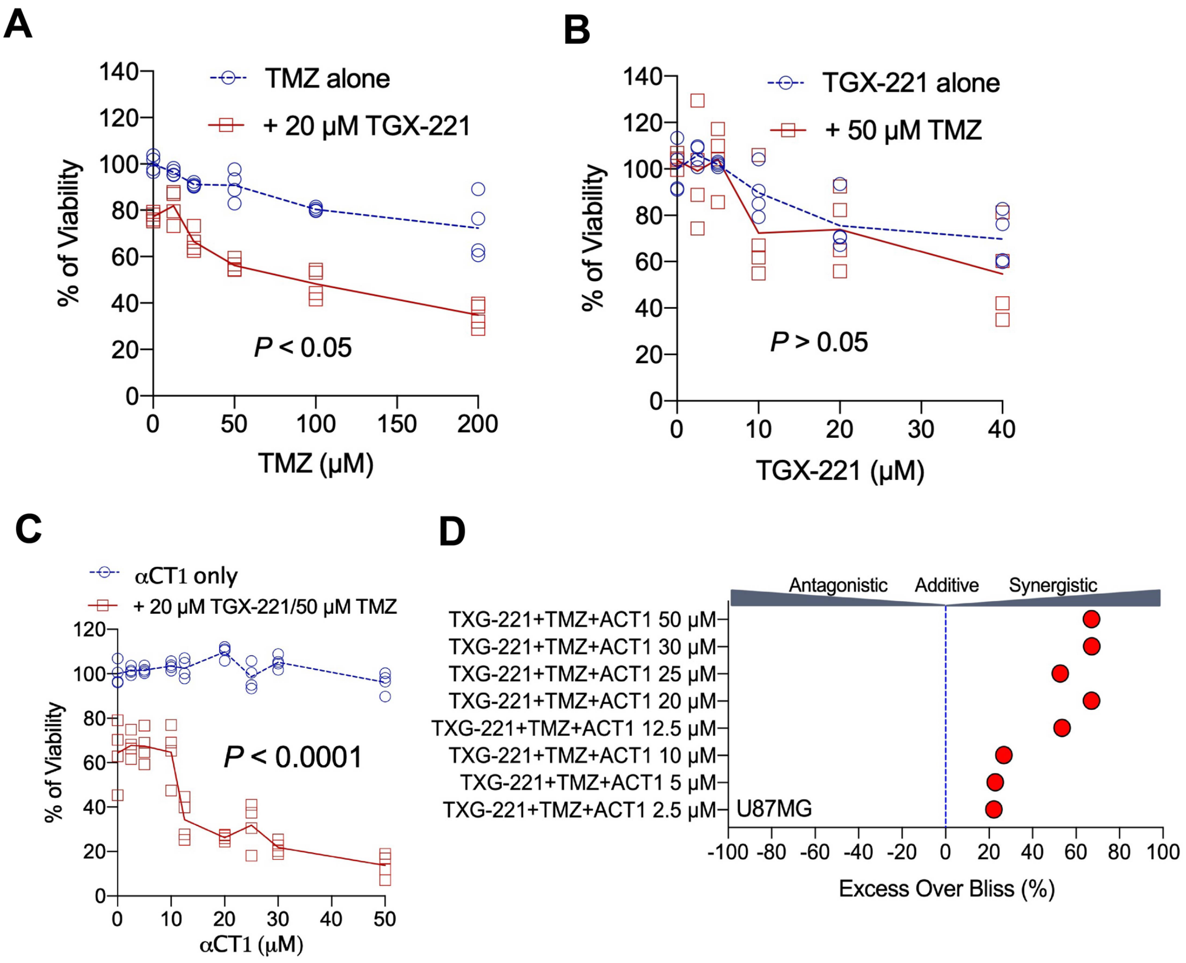

Supplemental Fig. S10. Optimization of αCT1, TGX-221 and TMZ in U87MG cells. (**A**) Combination of 20 μM TGX-221 and TMZ at various concentrations. U87MG cells were treated with drug combinations as indicated for 6 days. Cell viability was determined using the MTS viability assay. The vehicle DMSO was the control and set as 100%. Treated cells were normalized to DMSO-treated cells. (**B**) Combination of 50 μM TMZ and TGX-221 at various concentrations. (**C**) Combination of 20 μM TGX-221/50 μM TMZ and αCT1 at different concentrations. (**D**) Scores of Excess Over Bliss in **C** calculated using the Bliss Independence model. One-way ANOVA and student *t* test were used to determine statistical significance. Drug combinations with strong synergistic effect were marked in red.

Supplemental Fig. S11

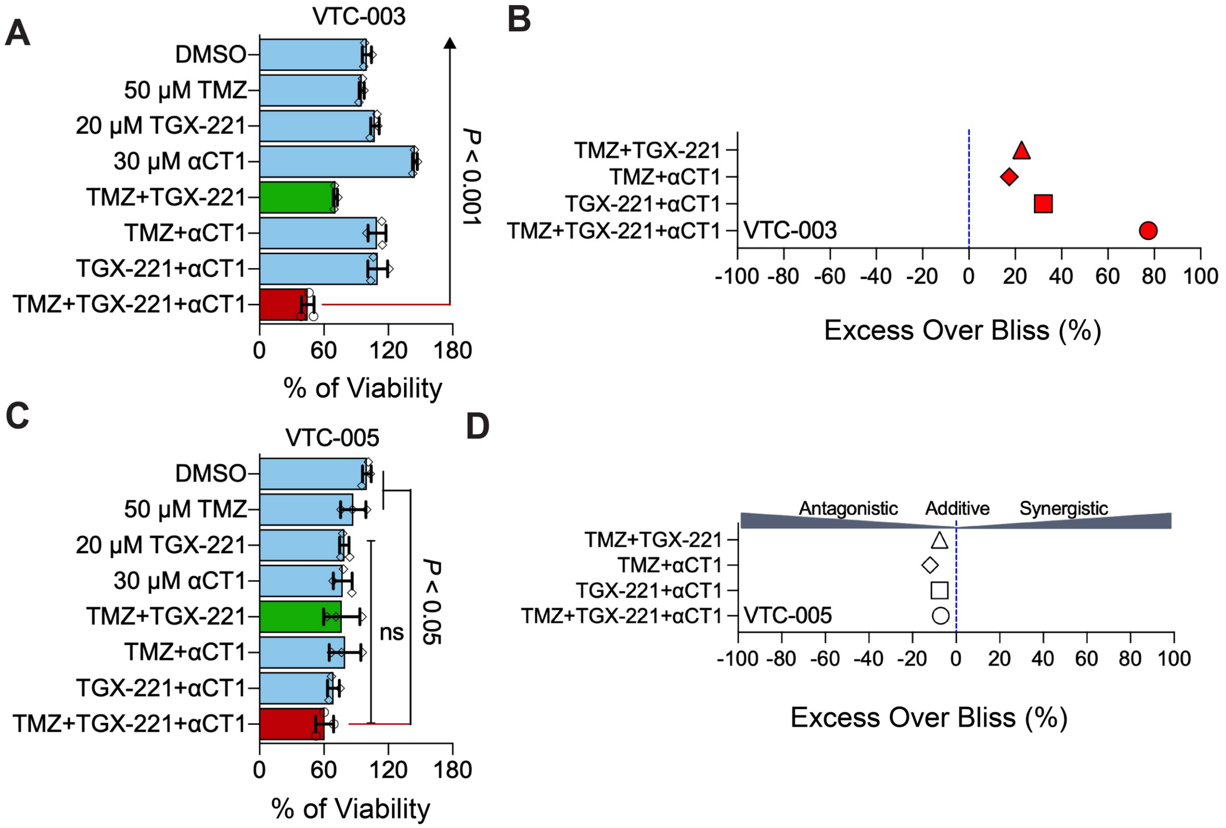

Supplemental Fig. S11. The αCT1/TGX combo in VTC-003 and VTC-005. (**A**) Viability of VTC-003 cells treated with different drug combinations for 6 days. Cell viability was determined using the MTS viability assay. (**B**) Scores of Excess Over Bliss of VTC-003 calculated using the Bliss Independence model. (**C**) Viability of VTC-005 cells treated with different drug combinations. (**D**) Scores of Excess Over Bliss of VTC-005 cells calculated using the Bliss Independence model. One-way ANOVA or student *t* test were used to determine statistical significance. Drug combinations with strong synergistic effect were marked in red.

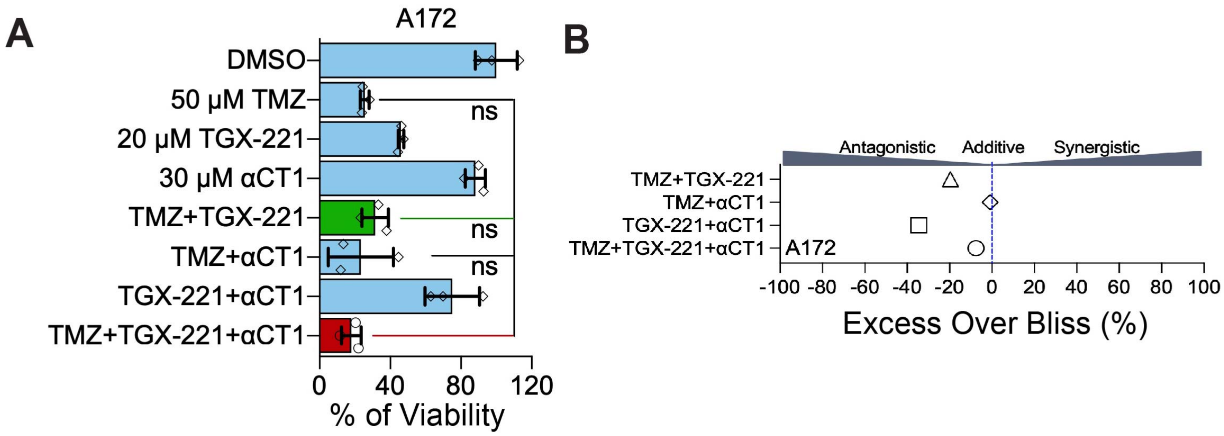

Supplemental Fig. S12. The αCT1/TGX combo in A172. (**A**) Viability of A172 cells treated with different drug combinations for 6 days. Cell viability was determined using the MTS viability assay. (**B**) Scores of Excess Over Bliss calculated using the Bliss Independence model. One-way ANOVA or student *t* test were used to determine statistical significance.

Supplemental Fig. S13

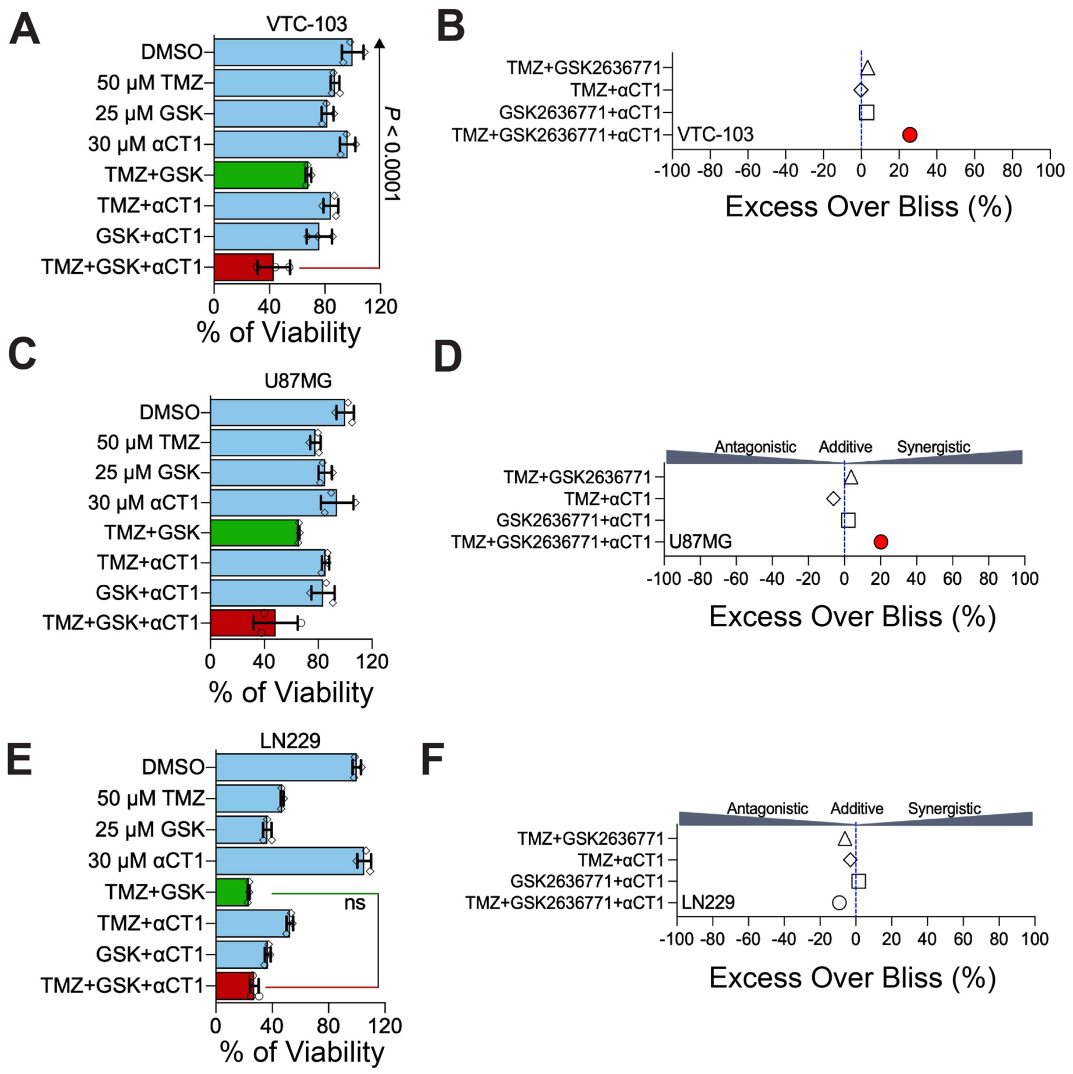

Supplemental Fig. S13. A combination of αCT1 and GSK2636771 overcomes TMZ resistance. The effect of the αCT1/GSK/TMZ combo in VTC-103 (**A**), U87MG (**C**), and LN229 (**E**) cells. Cells were treated with 50 µM TMZ, 25 µM GSK2636771, and/or 30 µM αCT1 including single agents, double combinations and the αCT1/GSK/TMZ combo. Excess Over Bliss scores of drug combinations in VTC-103 (**B**), U87MG (**D**), and LN229 (**E**) cells were calculated using the Bliss Independence mode. One-way ANOVA with Dunnett test for correction of multiple comparisons or Student's *t* test was used to determine statistical significance. ns: not significant. Drug combinations with strong synergistic effect were marked in red.

Supplemental Fig. S14

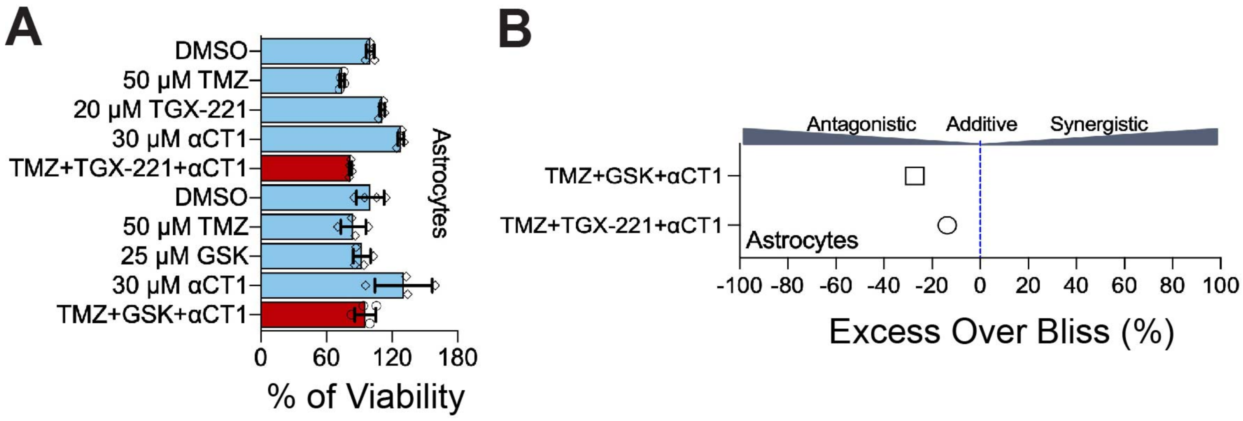

Supplemental Fig. S14. A combination of αCT1 and TGX-221 or GSK2636771 in astrocytes. (**A**) The effect of the αCT1/GSK/TMZ or αCT1/TGX-221/TMZ combo in astrocytes. Cells were treated with 50 µM TMZ, 30 µM αCT1, and/or 25 µM GSK2636771 or 20 µM TGX-221. (**B**) Excess Over Bliss scores of drug combinations in astrocytes cells were calculated using the Bliss Independence mode.
