# Supplementary material for: Connexin 43 confers chemoresistance through activating PI3K
Source: Oncogenesis. 2022 Jan 12;11(1):2. doi: 10.1038/s41389-022-00378-7 (PMC8755794; doi:10.1038/s41389-022-00378-7)

Supplemental Data-2

Connexin 43 confers chemoresistance through activating PI3K

Kevin J Pridham, Farah Shah, Kasen R Hutchings, Kevin L Sheng, Sujuan Guo, Min Liu, Pratik Kanabur, Samy Lamouille, Gabrielle Lewis, Marc Morales, Jane Jourdan, Christina L Grek, Gautam G Ghatnekar, Robin Varghese, Deborah F Kelly, Robert G Gourdie, and Zhi Sheng

Whole images of immunoblots

Figure 3 full images of immunoblotts

Figure 3A/U87MG

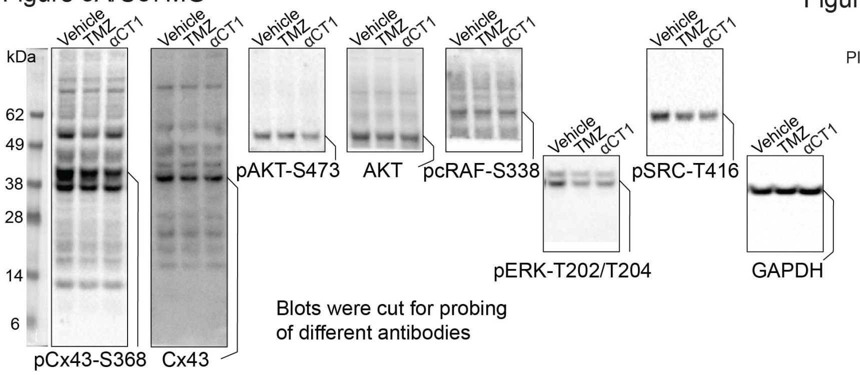

Figure 3G

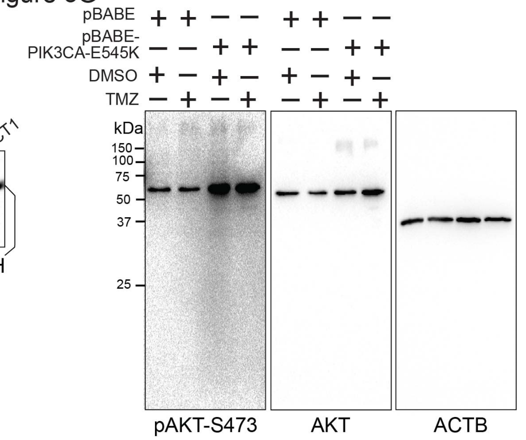

Figure 3B

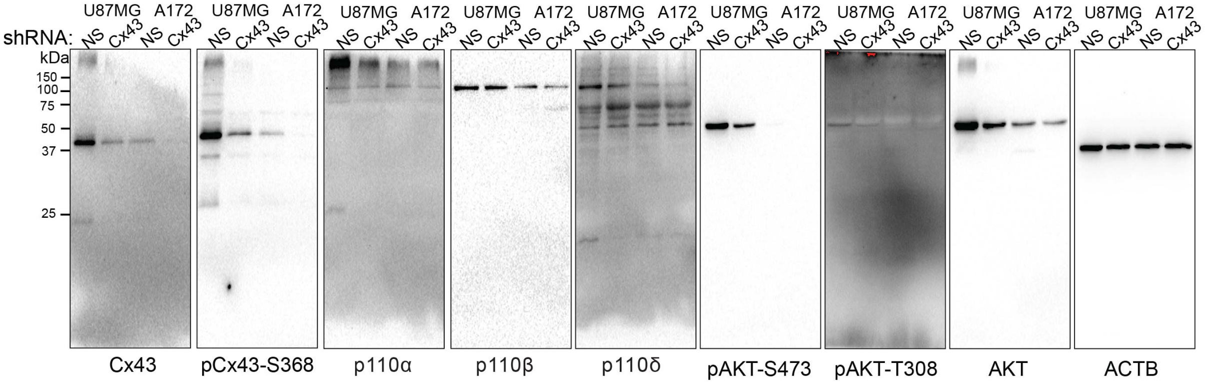

Figure 3C

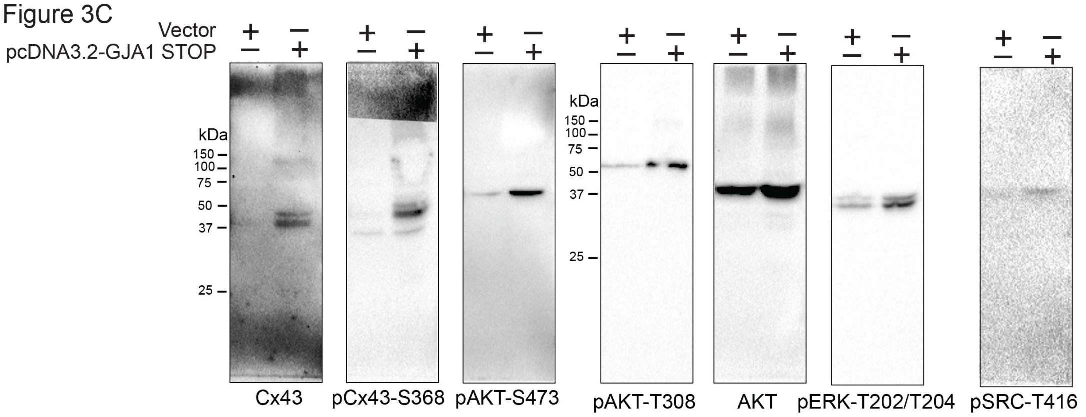

Figure 4 full images of immunoblotts

Figure 4A/U87MG

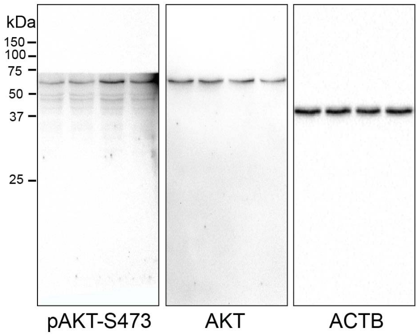

Figure 4L

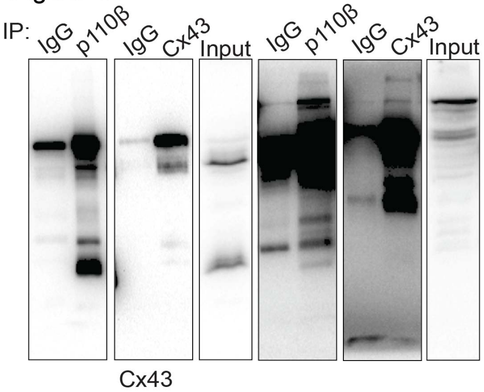

Figure 4M

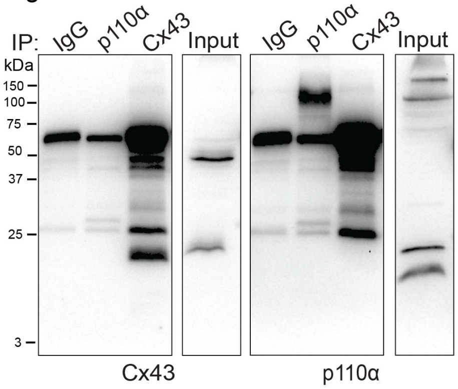

Figure 4N

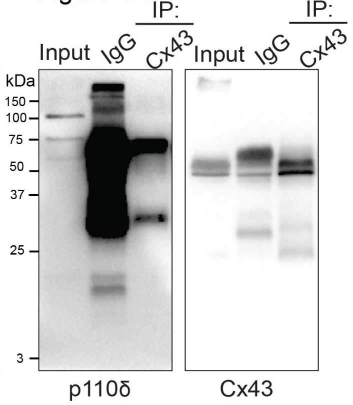

Figure 4O

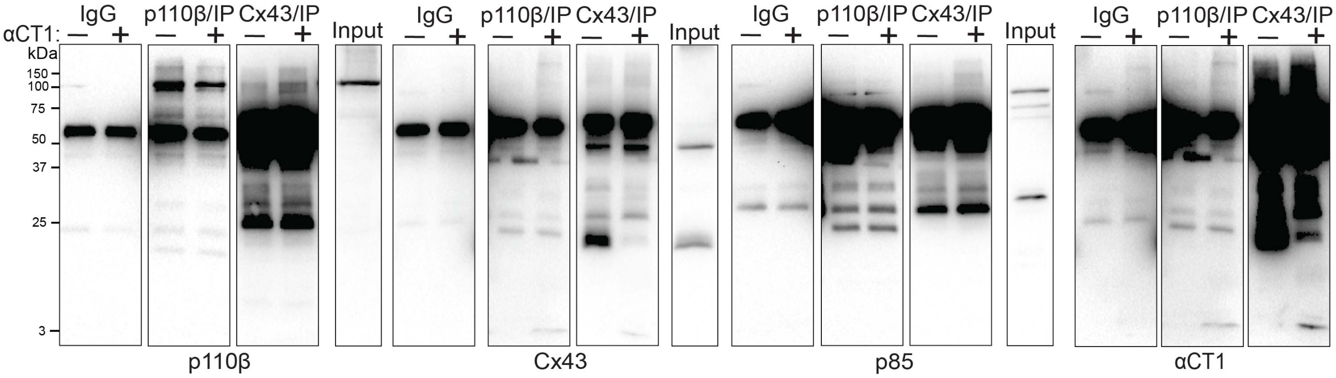

Figure5 full images of immunoblotts

Figure 5I

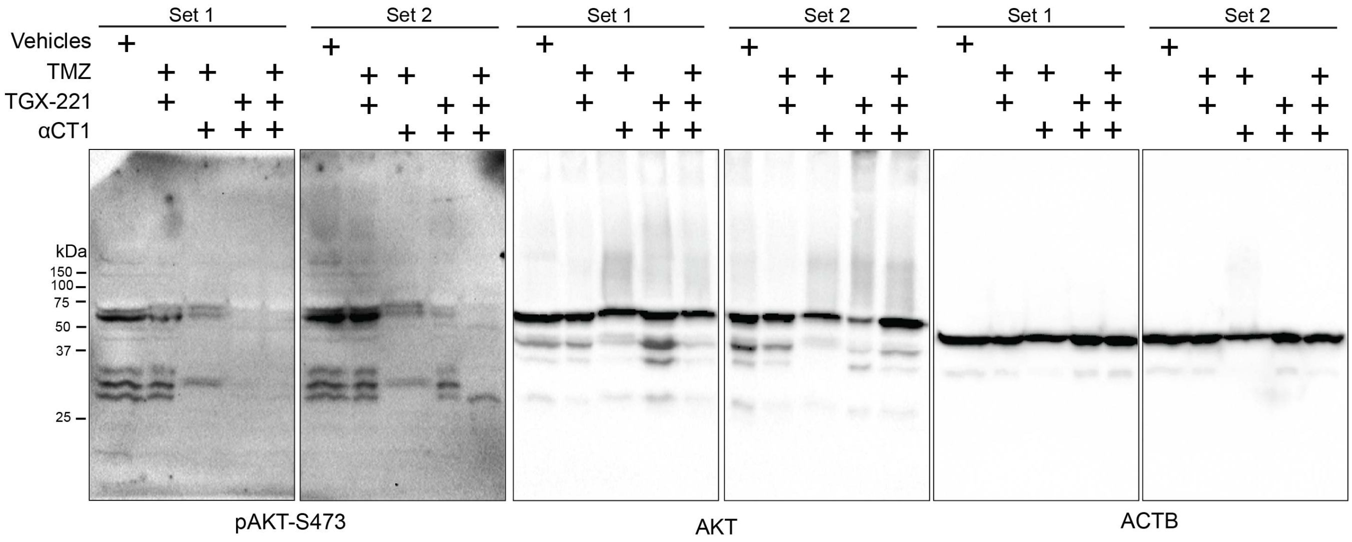

Supplement: Supplementary file 2 — Supplemental data-whole blot images [file 41389_2022_378_MOESM2_ESM.pdf]
